# Hook, Line, and Sinker! Spectroscopic Studies of Bi-Modular Mono- and Bis-1,8-naphthalimide-Ru(bpy)_3_-conjugates as DNA “Light Switches”

**DOI:** 10.1021/acs.inorgchem.2c00064

**Published:** 2022-07-25

**Authors:** Gary J. Ryan, Thorfinnur Gunnlaugsson, Susan J. Quinn

**Affiliations:** † School of Chemistry, Trinity Biomedical Sciences Institute (TBSI), Trinity College Dublin, The University of Dublin, Dublin 2, Ireland; ‡ School of Chemistry, University College Dublin, Dublin 4, Ireland; § Synthesis and Solid State Pharmaceutical Centre (SSPC), Bernal Institute, Limerick V94 T9PX, Ireland

## Abstract

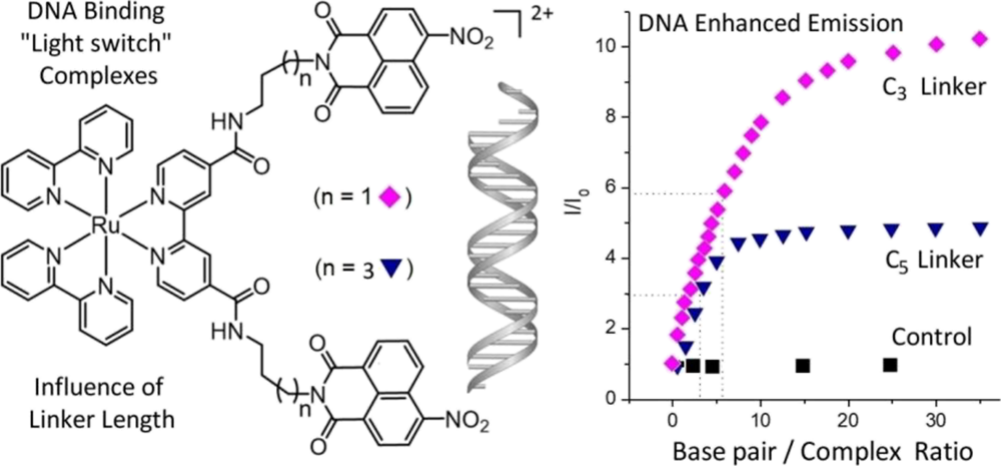

Bi-chromophoric ruthenium polypyridyl complexes comprising one or two nitro-1,8-naphthalimide groups are shown to be effective DNA binders with off–on light switching properties. The binding to DNA was investigated using a combination of studies such as UV–visible absorption and emission titrations, thermal denaturation, and circular dichroism spectroscopy. The DNA affinity was shown to be sensitive to both the linker length and the number of naphthalimides (one vs two) contained in these systems and binding constants ranging from 10^6^ to 10^7^ M^–1^ for salmon testes DNA. The strong DNA binding is attributed to the combination of naphthalimide intercalation and the electrostatic interaction of the ruthenium complex. Large emission enhancements from the metal to ligand charge transfer (MLCT) emission arising from the metal complex were observed upon DNA binding, which was attributed to the interruption of intramolecular electron transfer quenching processes. Moving the nitro substitution from the 4-position to the 3-position is found to result in modification of the DNA binding and the resulting optical properties. The off–on light switch phenomena reported demonstrate the potential of these complexes to act as DNA probes.

## Abstract 2

A family of bi-chromophoric ruthenium polypyridyl complexes comprising nitro-1,8-naphthalimide groups are shown to be effective DNA binders with off−on light switching properties attributed to disruption of MLCT quenching by the naphthalimide. The photophysical response is found to be sensitive to the number of naphthalimide units, the linker length, and the position of the nitro substituent. The off−on light switch phenomena reported demonstrate the potential of these complexes to act as DNA probes.

## Introduction

Ruthenium polypyridyl (Ru-polypyridyl) complexes have been extensively studied as photoreactive reagents, particularly for targeting DNA structures, as they possess a number of excellent tunable photophysical, photochemical, and redox properties, which may be exploited to probe their environment.^1−4^ Their DNA binding interactions have been extensively explored using spectroscopic methods,^5^ X-ray crystallography^6,7^ and biological investigations.^8,9^ These studies have shown how weak DNA binding exhibited by Ru(bpy)_3_^2+^ and Ru(phen)_3_^2+^ can be significantly improved through the use of extended polypyridyl ligands such as dipyrido[3,2-a:2′,3′-c]phenazine (dppz).^10,11^ The [Ru(bpy)_2_dppz]^2+^ and [Ru(phen)_2_dppz]^2+^ light switch complexes exhibit significantly enhanced emission upon DNA binding due to the sensitivity of the dipyridophenazine ligand to the solution environment.^10,11^ Furthermore, exchange of the bpy and phen auxiliary ligands with the 1,4,5,8-tetraazaphenanthrene (TAP) ligand yields an intercalating complex [Ru(TAP)_2_dppz]^2+^ capable of oxidizing guanine and forming a DNA adduct.^12,13^ In particular, ultrafast studies have contributed to our understanding of the mechanisms of photodamage through oxidation and/or photoadduct formation.^7,14−19^ Increasingly, their use and application in cellular imaging,^3,8,9,20−22^ and as light-activated theranostics and therapeutics^23−25^ are being explored. Ru(II) complexes are considered a promising class of PDT agents due to their reduced dark toxicity, photostability, spectroscopic properties, and significant DNA binding affinity.^2,3,9,26,27^ Notably, the thiophane containing TLD1433 developed by the McFarland group is the first ruthenium polypyridyl-based PDT agent to progress to clinical trials and is currently in a phase II study for treating nonmuscle invasive bladder cancer.^28^ There is also growing interest in developing Ru-polypyridyl as photoactivated antimicrobial agents.^29,30^ The study of Ru-polypyridyl systems in vitro and in vivo has been extensively featured in a number of recent reviews.^8,28,31^

Improved detection and binding of DNA by Ru(II) polypyridyl complexes can also be achieved through the attachment of DNA binding organic molecules.^32−35^ The organic chromophore can act to improve the binding affinity or in the case of an organic quencher, can act to signal the presence of DNA. An early example of the chromophore quencher system was a bifunctional Ru(II) complex appended to an aminoquinoline unit via a flexible linker. In the presence of DNA, the emission from the metal center was restored to varying degrees depending on the DNA sequence.^33^ A second example from the Turro group comprised a Ru(bpy)_3_^2+^ center tethered to an acceptor comprising a chain of two viologens.^34^ Organic 1,8-naphthalimides (**Nap**) are versatile molecules that can act as luminescent probes and photoreactive reagents for DNA through intercalation or grove binding^36,37^ and show promising anticancer properties.^38−40^ The highly tunable photophysical properties of the **Nap** structures have been widely explored for applications in chemical and biological sensing and imaging.^37,41^ We have previously reported the preparation of rigid Ru-polypyridyl 4-nitro- and 4-amino-1,8-naphthalimide conjugates that target DNA^42^ and shown that these complexes are readily internalized by cells by performing in depth bioprofiling.^43^ In a related study, we reported the DNA binding properties of mono- and bis-1,8-naphthalimide-ruthenium(II)-polypyridyl complexes conjugated through flexible linkers, which revealed greater photocleavage for the mono complex attributed to closer proximity of the Ru-center to DNA.^44^ In all cases, these complexes were found to show enhanced DNA binding, through multiple interactions, compared to the individual components. They also exhibit improved luminescent responses due to the interruption of the excited-state communication between the **Nap** and Ru-polypyridyl photophysical centers upon DNA binding. In related work, ruthenium organometallic N-heterocyclic carbene complexes with intercalating mono-naphthalimide^45^ and bis-naphthalimide ligands^46^ have been developed as anticancer and as antimicrobial agents.

**Figure 1:**
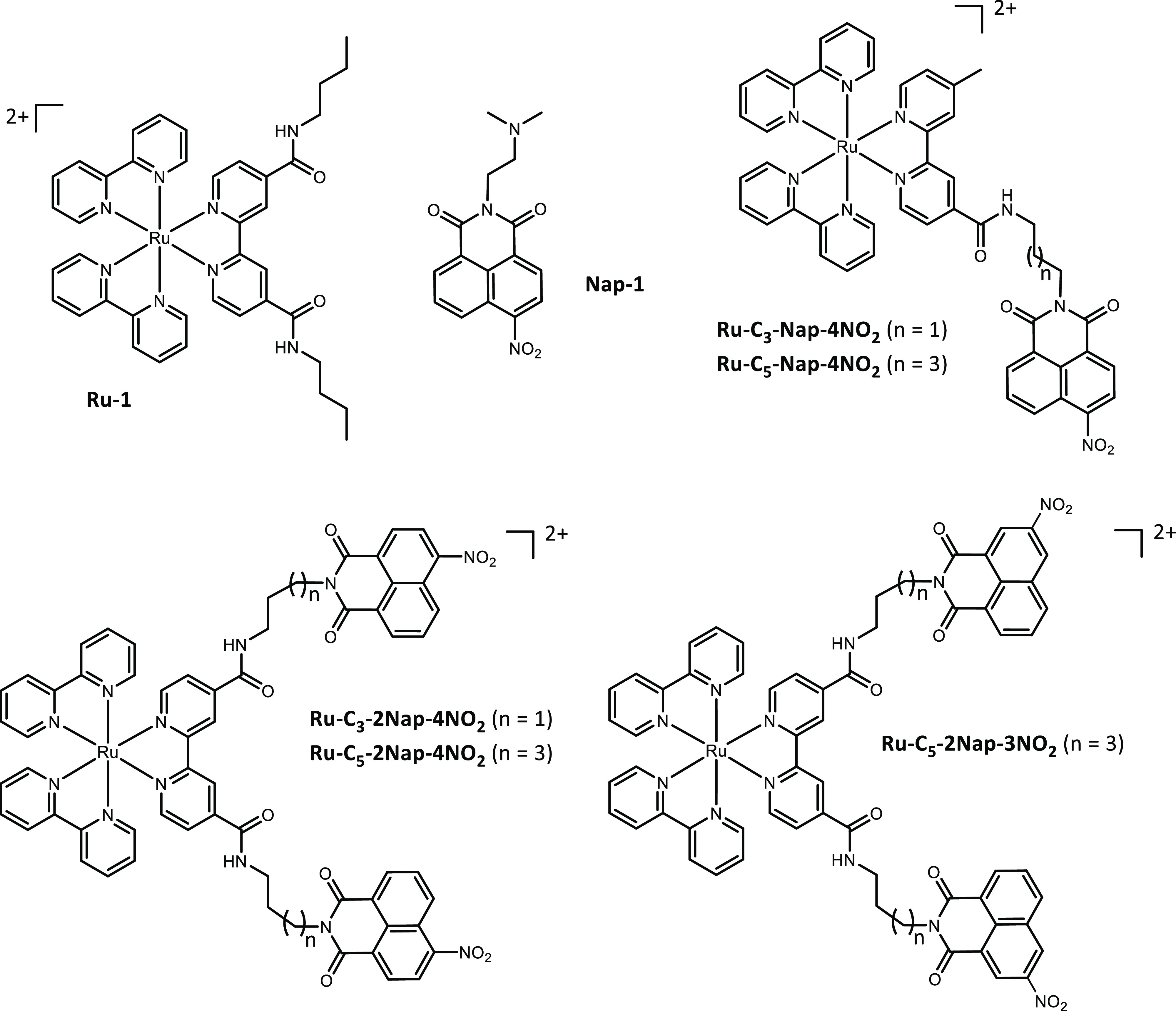
Family of five new bi-modular Ru(II) polypyridyl complexes and associated controls.

In this study, we report the photophysical properties of two families of chromophore quencher probes comprising ruthenium tris bipyridine complexes attached to 4-nitro-1,8-naphthalimide (quencher) units through a flexible linker. The complexes, referred to as **Ru-C_*x*_-Nap-4NO_2_** and **Ru-C_*x*_-2Nap-4NO_2_**, differ (a) in the number of 4-nitro-1,8-naphthalimide units appended to a bipyridine ligand (1 or 2) and (b) the length of the alkyl spacer (*x* = 3 or 5), see Figure 1. The difference in the linker length was chosen to investigate the influence of the ligand flexibility on the possible binding. The properties of the complexes are compared to those of the corresponding Ru-polypyridyl **Ru-1** and water-soluble 4-nitro-1,8-naphthalimide **Nap-1** controls and then profiled in the presence of DNA. The comparison of mono- and bis-naphthalimide systems is expected to reveal possible enhancement or the role of cooperative binding, where the potential intercalation of one of the naphthalimide units (strongly influenced by the electrostatic interaction of the Ru(II)-polypyridyl center) would facilitate the binding of the second naphthalimide unit.

The distinctive spectroscopic signals associated with each component are used to report on their local environment, and we demonstrate that these complexes are capable of intercalative DNA binding via the planar 4-nitro-1,8-naphthalimide group and via external binding through electrostatic interaction of the Ru(II) complex with the anionic backbone of DNA. Finally, we consider the impact of changing the nitro substitution from the 4-position to the 3-position in the bis-naphthalimide complex **Ru-C_5_-2Nap-3NO_2_** on its ability to act as a DNA probe. Overall, this study shows that binding interaction of the naphthalimide to DNA disrupts its ability to quench the Ru(II) emission and leads to an “off–on light-switch” like effect and that the degree of this effect can be tuned by adjusting the design parameters of the complex.

## Results and Discussion

### Design and Synthesis

**Scheme 1:**
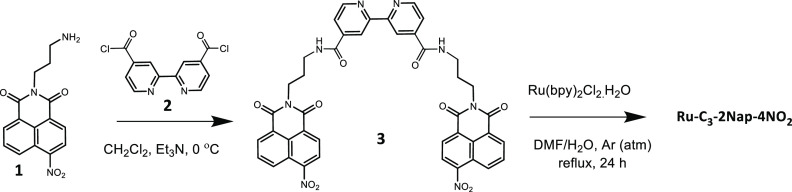
Synthesis of **Ru-C_3_-2Nap-4NO_2_** from the Naphthalimide **1** (Used as TFA Salt) and the Acid Chloride bpy **2**

The design of our Ru-polypyridyl-Nap complexes was in part inspired by the work of Le Pecq and co-workers, who have shown that bis-intercalation can occur for long chain adducts in which it is possible to incorporate two base pairs between the bound ligands, in a mode that follows the neighbor exclusion principle.^47^ The minimum chain length corresponding to this separation is 10.2 Å. However, similar studies by Wakelin and co-workers set this minimum value at 8.8 Å, which would give rise to a bis-intercalative mode with violation of the neighbor exclusion principle.^48^ When the linker is rigid or insufficiently long mono-intercalation results.^49^ The syntheses of the Ru(II)-complexes, including the control **Ru-1,** were generally achieved in a few steps from commercially available starting materials in good to medium yields for the final products. Details of the synthesis and characterization of these and the precursor ligands are given in the Experimental Section and the Supporting Information. As an example, an outline of the synthesis of **Ru-C_3_-2Nap-4NO_2_** is shown in Scheme 1. The first step involved the synthesis of the 4-nitro-1,8-naphthalimide bpy ligand structure **1** in three steps from the Boc-protected diamine and 4-nitro-1,8-napthalic anhydride.^44^ Deprotection using TFA and coupling with di-acid chloride of 2,2′-bipyridine **2** yielded the bis-1,8-naphthalimide-bipyridine ligand **3** (see ^1^H NMR in the Supporting Information Figure S1, and Figure S2 for the mono analogue), which were then reacted with Ru(bpy)_2_Cl_2_.2H_2_O under reflux conditions in DMF/H_2_O for 24 h to yield the desired tris Ru(II) complex **Ru-C_3_-2Nap-4NO_2_**.

**Figure 2:**
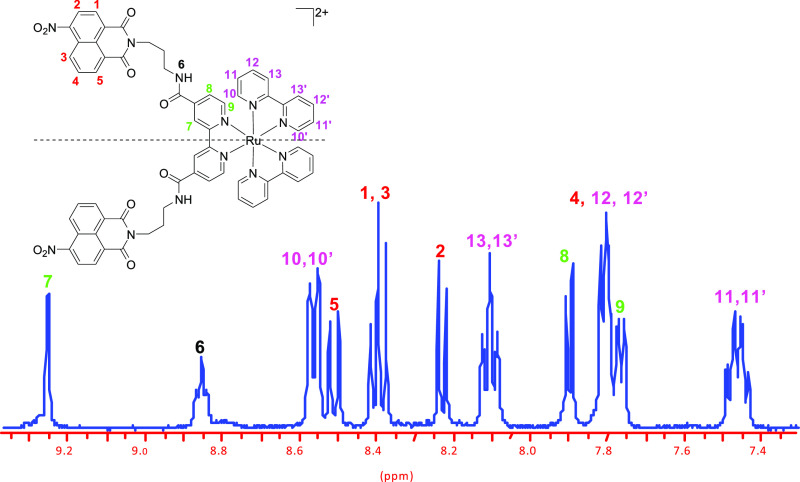
Assigned ^1^H NMR spectrum (CD_3_CN, 400 MHz) of **Ru-C_3_-2Nap-4NO_2_** showing the aromatic regions.

The complex was initially purified by silica column flash chromatography followed by subsequent size exclusion chromatography, using Sephadex LH-20 eluting with MeOH 100%. This treatment gave the desired product as a red solid in 52% yield. A partial ^1^H NMR spectrum of **Ru-C_3_-2Nap-4NO_2_** showing the aromatic regions is shown in Figure 2, demonstrating the C_2_ symmetry of the system (the corresponding ^1^H NMR spectrum of **Ru-C_3_-Nap-4NO_2_** is given for comparison in the Supporting Information, Figure S3). The same approach was used to prepare **Ru-C_5_-2Nap-3NO_2_**.

### Photophysical Properties of Ruthenium Complexes

**Figure 3:**
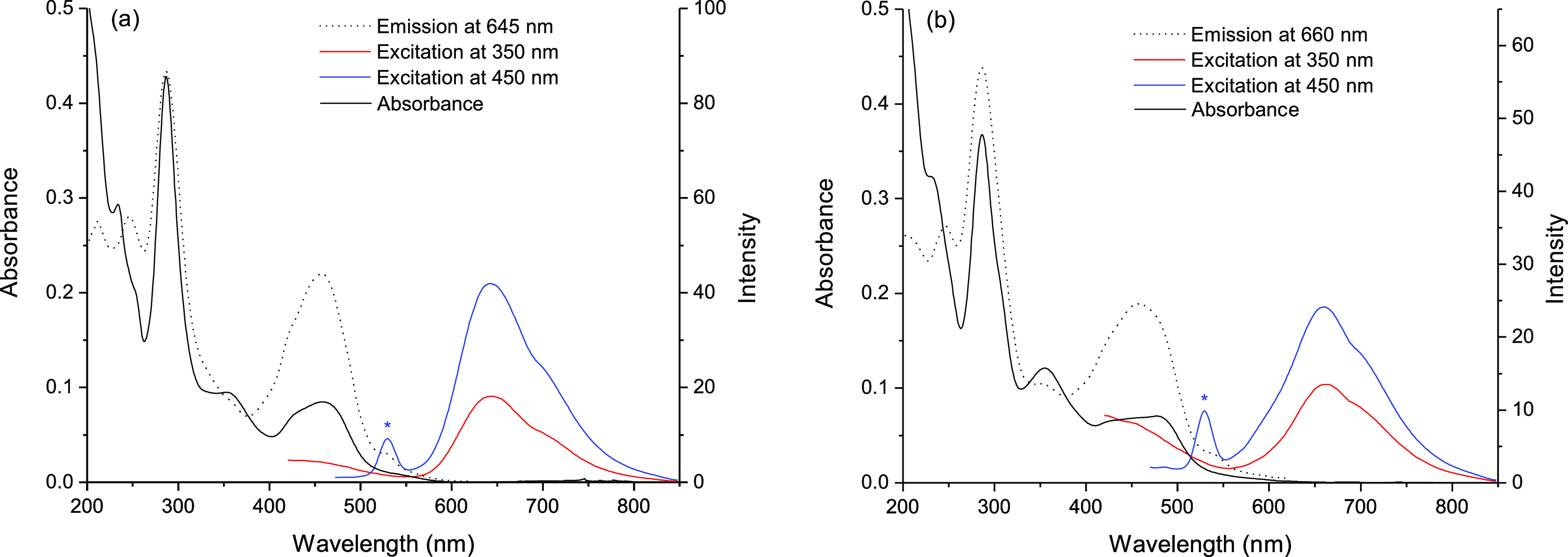
UV/Visible, excitation, and emission spectra of (a) **Ru-C_5_-Nap-4NO_2_** and (b) **Ru-C_5_-2Nap-4NO_2_** (both at 6.5 μM). The water Raman band is denoted by * in each case.

The UV/visible absorption spectra of the **Ru-C_*x*_-Nap-4NO_2_** and **Ru-C_*x*_-2Nap-4NO_2_** complexes exhibited bands characteristic of Ru(II) polypyridyl and naphthalimide chromophores. The UV/visible absorption spectra of aqueous solutions of **Ru-C_5_-Nap-4NO_2_**, and **Ru-C_5_-2Nap-4NO_2_** are shown in Figure 3. For both, the band at 285 nm is attributed to π–π* intraligand (IL) transitions of the bipyridine and the band at 350 nm to the π–π* transition of the naphthalimide. A broadened metal to ligand charge transfer (MLCT) transition band is observed at 456 nm for **Ru-C_5_-Nap-4NO_2_** and at 477 nm for **Ru-2C_5_-Nap-4NO_2_** with an intermediate effect being observed for **Ru-C_5_-Nap-4NO_2_**. The red shift in the MLCT band compared to [Ru(bpy)_3_]^2+^ is due to the amide-substituted bipyridine ligands, which yields bipyridine ligands with more positive reduction potentials.^50^ Charge transfer to the nonsubstituted ligand contributes mainly in the 450–460 nm region, and the lower energy transition to the amide-substituted ligand in the 490–500 nm region.

The degree of intramolecular stacking of the naphthalimides in the complexes was investigated by comparing the absorption spectrum to the predicted spectrum for a complex with no stacking obtained by addition of the controls **Ru-1** and **Nap-1**. For **Ru-C_5_-2Nap-4NO_2_**, the MLCT region for the sample and predicted spectrum is in close agreement, see Figure S4. However, the naphthalimide absorbance is significantly ca. 30% less than that observed for the calculated species. This hypochromicity suggests a degree of intramolecular stacking of Nap moieties in **Ru-C_5_-2Nap-4NO_2_**. Such interaction could be expected due to the flexible nature of the linker and the propensity of 1,8-naphthalimides to stack in polar solution, due to their planar and hydrophobic nature.^51^ A similar effect was previously observed for the related unsubstituted **Ru-C_5_-2Nap** complex where intramolecular stacking interactions was found to cause a 47% decrease in the absorption of the naphthalimide absorbance at 345 nm.^44^ The reduced effect observed for **Ru-C_5_-2Nap-4NO_2_** compared to **Ru-C_5_-2Nap** suggests that the presence of the nitro substituents reduces the degree of intramolecular stacking interactions.

It has previously been shown by transient resonance Raman spectroscopy that rapid intramolecular charge transfer occurs upon light absorption systems ncorporating different ligands around the metal center, which yields a triplet MLCT state in which the excited state is localized on the ligand having the most positive reduction potential.^52^ Thus emission is expected to arise solely from the excited state where the electron is localized on the amide ligand. Excitation of the mono-naphthalimide **Ru-C_5_-Nap-4NO_2_** complex at 450 nm resulted in a single MLCT emission band centered at 645 nm, which is reflected in the excitation spectrum, see Figure 3a. The MLCT-based emission is red-shifted (665 nm) in the case of **Ru-C_5_-2Nap-4NO_2_** (see Figure 3a) due to the second amide on the bipyridine ligand. Excitation of the **Nap-1** control at 350 nm yields weak emission at ca. 420 nm, while excitation of the Ru-Nap complexes at 350 nm results in weak emission from both the **Nap** and comparably stronger emission from the metal complex. The MLCT-based emission observed for the Ru-Nap complexes in aerated solution was found to be significantly less than that observed for **Ru-1**, which is reflected in the low quantum yields (QYs) (≤0.001) obtained, see Table 1. The impact of the nitro-substituted naphthalimides on the QY is in contrast to what was previously reported for the structurally similar unsubstituted **Ru-C_5_-2Nap** complex, whose QY (0.014) in aerated solution was found to be comparable to that of the **Ru-1** control complex.^44^ The lower QY observed in the nitro-substituted systems is attributed to interaction between the Nap moieties and the MLCT triplet state. Similar phenomena have been observed for Ru(II)-aminoquinoline^32^ and Ru(II)-viologen^34^ conjugates and are attributed to electron transfer processes between the Ru(II) center (chromophore) and the organic moiety (quencher). The excited state of the metal complex can act as a good electron donor or electron acceptor. While the presence of the substituted **Ru-C_5_-2Nap** naphthalimide was not observed to impact the QY of emission, the presence of the 4-nitro substituent results in a more electron-deficient naphthalene ring,^53^ which may accept an electron from the excited state of the metal complex and the associated lowering of the QY. The magnitude of the QY suggests that the excited state of the metal complex is efficiently quenched by electron transfer to the 1,8-naphthalimide moiety. As such Ru-Nap has potential to act as chromophore-quencher probes of DNA, where binding to DNA changes the environment of the naphthalimide so it no longer can participate in quenching and thus restores the luminescence, yielding an off–on light switch effect.^54^ A summary of the spectroscopic properties is tabulated in Table 1.

**Table 1:** Spectroscopic Properties of Complexes in 10 mM Phosphate Buffer, pH 7 at 298 K^a^

complex	λ_max_ (nm) [ε (M^–1^ cm^–1^)]			λ_em_ (nm)	Φ_f_ (± 10%)
	π–π* IL	π–π* Nap	MLCT		
**Ru-1**	286 [55,210]		475 [10,900]	670	0.014
**Ru-C_3_-Nap-4NO_2_**	286 [65,600]	351 [14,500]	458 [13,300]	645	0.001
**Ru-C_5_-Nap-4NO_2_**	286 [73,900]	351 [14,900]	456 [13,700]	645	0.001
**Ru-C_3_-2Nap-4NO_2_**	286 [60,900]	356 [21,300]	477 [12,700]	665	< 0.001
**Ru-C_5_-2Nap-4NO_2_**	286 [55,800]	356 [20,100]	477 [11,100]	665	< 0.001

a All the data were fully reproducible over several measurements. Φ_f_ measured in air-saturated solution.

### DNA Binding Interactions

#### Visible Absorption Studies

**Figure 4:**
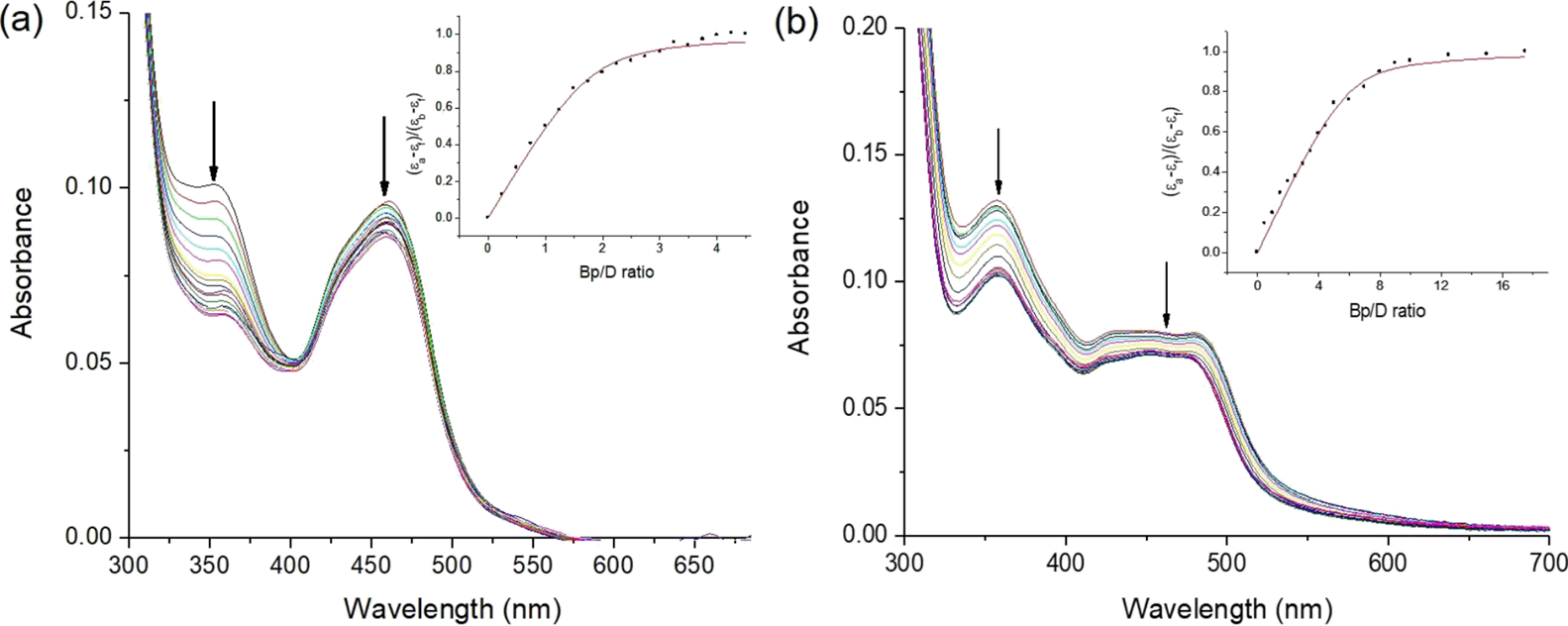
Changes in the UV/visible absorption spectrum of (a) **Ru-C_5_-Nap-4NO_2_** (6.5 μM) upon addition of st-DNA (0–29.25 μM base pair) and (b) **Ru-C_5_-2Nap-4NO_2_** (6.5 μM) upon addition of st-DNA (0–130 μM) in 10 mM phosphate buffer, at pH 7. Inset: Plot of (ε_a_ – ε_f_)/(ε_b_ – ε_f_) vs [DNA] (expressed as Bp/D ratio, where Bp = base pair and D = complex) and the corresponding nonlinear fit.

The interaction of the four **Ru-Nap-4NO_2_** complexes with st-DNA, which is as a source of random sequence DNA comprising approximately 2000 base pairs with a GC content of 41.2%, was first examined by performing additions of the DNA to solutions of each complex, see Figure 4 and Figures S5 and S6. In all cases, modest hypochromism (10%) was observed for the MLCT band at 450 nm and is similar to that observed for the control complex, **Ru-1** and previously reported for [Ru(bpy)_3_]^2+^, which is likely due to electrostatic interactions between the complex and the phosphate backbone of DNA.^55^ In contrast, the addition of DNA resulted in 20–35% hypochromism of the naphthalimide π–π* transition at 350 nm, which is characteristic of DNA intercalation.^37^ The greatest effect (35% hypochromism and 4 nm bathochromic shift) was observed for the mono-pentyl **Ru-C_5_-Nap-4NO_2_** complex. In comparison, 20% hypochromic shift was observed for the mono-propyl **Ru-C_3_-Nap-4NO_2_**, which indicates the influence of the linker length on the binding interaction (see Figure S5). In the case of the bis-naphthalimide systems, a slightly greater effect was found for **Ru-C_5_-2Nap-4NO_2_** (27%) compared to the **Ru-C_3_-2Nap-4NO_2_** (24%) complex; see in Figures 4b and S6. The magnitudes of the changes in the naphthalimide and MLCT bands were found to be less than those previously observed for the related unsubstituted complexes **Ru-C_5_-Nap** (Nap 37%, MLCT 16%) and **Ru-C_5_-2Nap** (Nap 56%, MLCT 41%), which indicates that the presence of the nitro groups significantly impacts the response of the complexes to the presence of DNA.^44^

Binding constants (*K*) of 10^6^ M^–1^ were determined from analysis of the changes at 350 nm using the Bard model,^56^ see Table 2. A similar binding site size (n base pairs) of ∼1.65 was determined for the mono-naphthalimides, which was found to increase to ∼3.5 base pairs for the bis-propyl **Ru-C_3_-2Nap-4NO_2_** complex and to 6 base pairs for the bis pentyl **Ru-C_5_-2Nap-4NO_2_** complex. The large binding site size for **Ru-C_5_-2Nap-4NO_2_** suggests the possible interaction of both naphthalimide components of the complex with DNA. The extent of hypochromism observed for the bimodular complexes above is similar to that previously observed for a rigidly appended 4-nitro-naphthalimide to a [Ru(bpy)_3_]^2+^.^42^ There are a number of factors that may impact the binding interactions, which include the interplay between steric interactions, hydrophobicity, and flexibility.^57−59^ Generally, bis-intercalating species are expected to display DNA binding affinity more than an order of magnitude greater than the corresponding mono-intercalating species. However, here the structurally different complexes display binding constants of a similar order. Yet this does not mean that the complexes bind in the same way; for example, Becker et al. observed similar binding constants for anthracene systems, which were found to bind in different modes.^60^ In any system, the binding parameters represent an average that results from possible different binding geometries and a number of possible binding modes are available for these the flexible multicomponent complexes. The contribution of different binding modes may contribute to the unusual observation of the slightly weaker binding constant for the bis system **Ru-C_5_-2Nap-4NO_2_** compared to the other complexes, which also has a significantly larger binding site size than the other complexes. It is also necessary to consider that intramolecular electronic coupling between the chromophores influences the steady-state absorption, see Figure S4. Therefore, the changes in the spectra are expected to be due to a combination of decreased intramolecular coupling between chromophores and increased intermolecular interactions with DNA. The interplay of these phenomena makes extraction of the binding constants more challenging, especially in the case of the bis-naphthalimide systems. Applying the same data treatment, our previous study on the structurally related unsubstituted naphthalimide C_5_ linker complexes revealed binding constants of 9.6 × 10^6^ (± 1.0) M^–1^ for the mono complex (**Ru-C_5_-Nap)** and 1.5 × 10^7^ (± 0.5) M^–1^ for the bis complex (**Ru-C_5_-2Nap**), which indicates that the presence of the nitro substituent may reduce the binding affinity.^44^

**Table 2:** DNA Binding Parameters from Fits to Absorbance Data

complex	λ (nap) hypochromism	λ (MLCT) hypochromism	binding constant *K* (M^–1^)	binding site size *n* (base pairs)	*R*^2^
**Ru-1**		9%			
**Ru-C_3_-Nap-4NO_2_**	20%	9%	3.2 × 10^6^ (± 1.0)	1.6 (± 0.1)	0.99
**Ru-C_5_-Nap-4NO_2_**	35%	10%	4.6 × 10^6^ (± 1.0)	1.7 (± 0.1)	0.99
**Ru-C_3_-2Nap-4NO_2_**	24%	10%	9.2 × 10^6^ (± 4.0)	3.5 (± 0.1)	0.99
**Ru-C_5_-2Nap-4NO_2_**	27%	10%	2.8 × 10^6^ (± 0.8)	6.0 (± 0.3)	0.99

### Emission Studies

**Figure 5:**
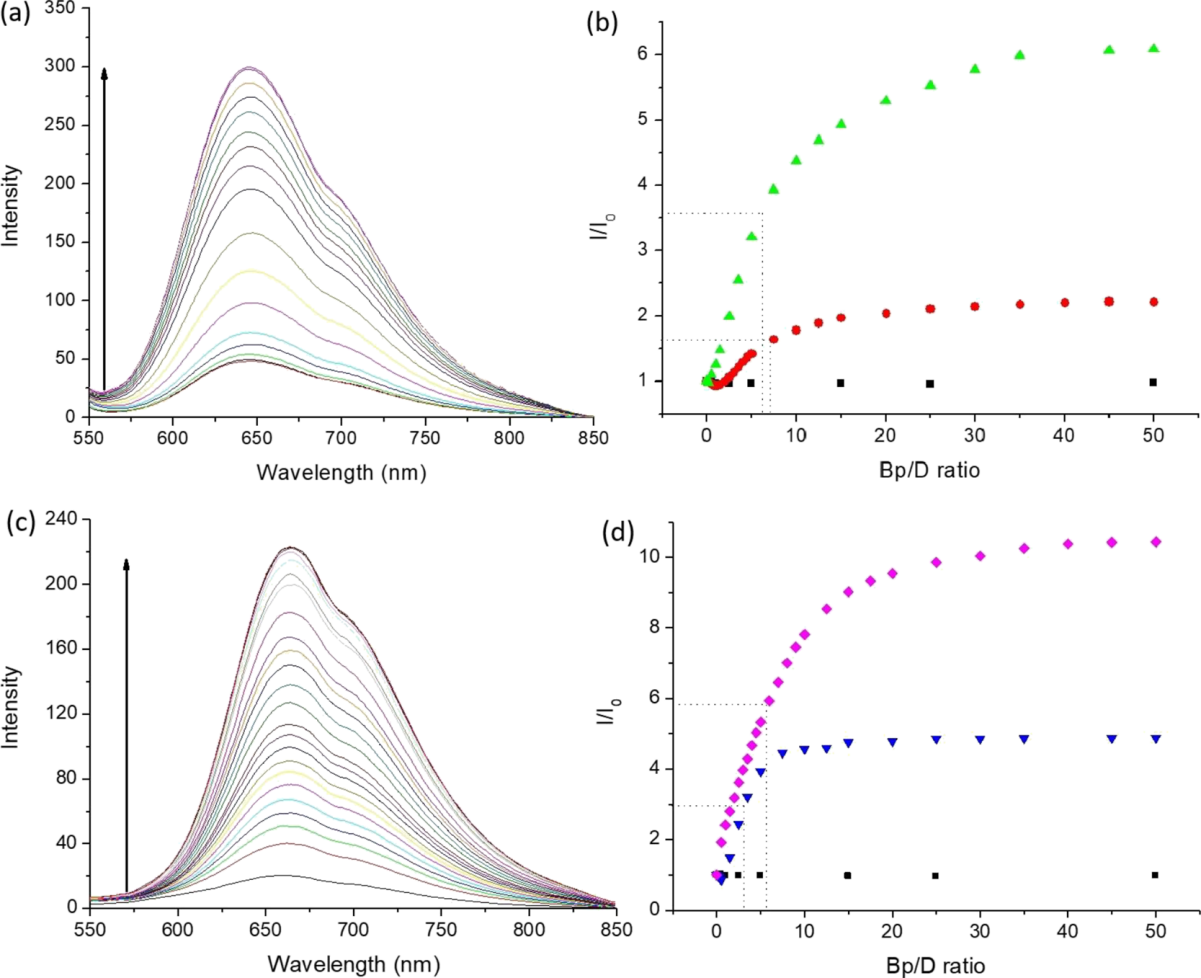
Changes in the MLCT emission of (a) **Ru-C_5_-Nap-4NO_2_** and (c) **Ru-C_5_-2Nap-4NO_2_** upon addition of st-DNA (0–325 μM base pairs). The relative change in integrated emission intensity of (b) **Ru-C_3_-Nap-4NO_2_** (red circle), **Ru-C_5_-Nap-4NO_2_** (green upward pointing triangle), and **Ru-1** (solid box) and (d) **Ru-C_3_-2Nap-4NO_2_** (blue downward pointing triangle), **Ru-C_5_-2Nap-4NO_2_** (purple diamond), and **Ru-1** (solid box) upon addition of st-DNA. All in 10 mM phosphate buffer, at pH 7, expressed as the Bp/D ratio based on the complex concentration of 6.5 μM (λ_ex_ 450 nm).

The potential of the Ru(II)-Nap complexes to act as chromophore-quencher probes was studied by examining the ability of DNA binding to disrupt intermolecular quenching of the Ru(II) polypyridyl triplet^3^ MLCT state by the appended naphthalimide to yield an off–on light switch effect. The addition of st-DNA to a solution of the flexible monopentyl **Ru-C_5_-Nap-4NO_2_** in 10 mM phosphate buffer resulted in a ca. 6-fold increase in the emission (Figure 5a,b) with the half point in emission enhancement reached at a Bp/D ratio of 6.7. The enhancement was found to be significantly less for the monopropyl **Ru-C_3_-Nap-4NO_2_**, which saw a 2-fold increase upon that addition of similar DNA equivalents. The difference in enhancement is attributed to the greater intramolecular quenching in the **Ru-C_5_-Nap-4NO_2_** prior to the addition of DNA. Not surprisingly, no emission enhancement was observed for **Ru-1**, which clearly demonstrates the importance of the naphthalimide structures in both the binding and the modulation of the concomitant photophysical properties.

For the bis-naphthalimides, the linker length was also found to influence the emission enhancement upon DNA binding, with a ca. 5-fold increase for the propyl **Ru-C_3_-2Nap-4NO_2_** compared to a ca. 11-fold enhancement for the pentyl **Ru-C_5_-2Nap-4NO_2_**, see Figure 5. In addition, the titration profiles of the bis-naphthalimide systems showed a sharper increase in the enhancement, which is reflected in the lower equivalents of DNA base pairs (Bp) required to reach the half point in emission enhancement, see Table 3 and Figure S7. The broader profile of emission enhancement observed for the mono-naphthalimide systems is likely to reflect the greater degree of flexibility in these systems. It should be noted that in a structurally simple Ru(II) complex such as [Ru(phen)_3_]^2+^, emission enhancement upon DNA binding is attributed to shielding of the Ru(II) complex core from quenching by oxygen and solvent molecules and reduced vibrational deactivation of the excited state due to rigidification of the bound complex. This mechanism may play a small role in the emission enhancement for observed for the complexes, in which the Nap moiety tightly binds DNA, and in doing so locks the Ru(II) center in place in the protected environment of the DNA grooves, thereby shielding it from quenching. The overall results are summarized in Table 3.

**Table 3:** Emission Titration Data for Complexes with st-DNA^a^

complex	10 mM phosphate buffer		10 mM phosphate buffer + 50 mM NaCl		10 mM phosphate buffer + 100 mM NaCl	
	*E*_f_	Bp/D ratio *F*_(obs)_ = 0.5F_(final)_	*E*_f_	Bp/D ratio *F*_(obs)_ = 0.5F_(final)_	*E*_f_	Bp/D ratio *F*_(obs)_ = 0.5F_(final)_
**Ru-C_3_-Nap-4NO_2_**	2.1	7.8	1.9 (↓10%)	10.5	1.8 (↓15%)	13.9
**Ru-C_5_-Nap-4NO_2_**	6.1	6.7	5.5 (↓10%)	9.4	4.0 (↓34%)	16.1
**Ru-C_3_-2Nap-4NO_2_**	4.9	3.2	4.3 (↓12%)	4.3	3.8 (↓23%)	8.6
**Ru-C_5_-2Nap-4NO_2_**	10.7	5.5	7.6 (↓29%)	11.5	6.3 (↓42%)	17.0

a Enhancement factor *E*_f_.

The off–on light switch effect was next tested under more biologically relevant conditions by the addition of 50 mM and 100 mM NaCl. This resulted in a decrease amount of emission enhancement and the addition of greater equivalents of DNA, see Figures S8 and S9 and Table 3. These observations are taken to reflect the screening of the electrostatic component of the binding interaction of the charged species. The **Ru-C_5_-2Nap*-*4NO_2_** complex exhibited the greatest enhancement across all conditions with a ca. 6-fold increase found at 100 mM NaCl, see Figure S9. Notably the enhancement properties of the propyl systems were found to be less affected by the increase in ionic strength, see Figures S8 and S9, which suggests that there are less electrostatic contributions to their DNA binding with a 4-fold increase in emission still observed for **Ru-C_3_-Nap-4NO_2_** in 100 mM NaCl.

### Thermal Denaturation Studies

**Figure 6:**
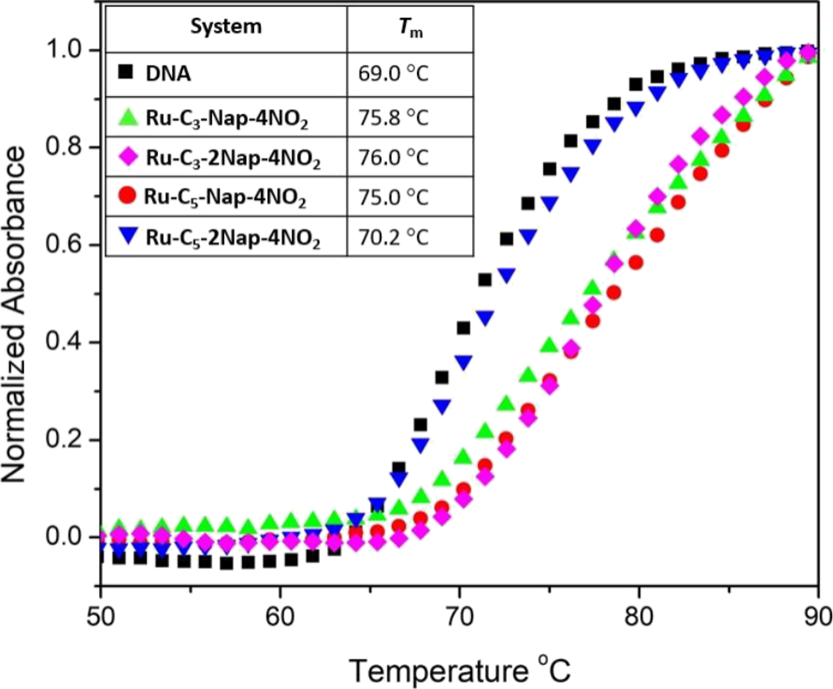
Thermal denaturation curves of 150 μM st-DNA in 10 mM phosphate buffer, at pH 7, in the absence of **Ru-C_*x*_-Nap-4NO_2_** and **Ru-C_*x*_-2Nap-4NO_2_** complexes all at a Bp/D ratio of 5.

The ability of the complexes to stabilize the DNA structure was investigated by performing UV thermal melting studies, from which the helix-coil transition temperature (*T*_m_) was determined.^61^ In the absence of Ru(II)-complexes, the melting point of st-DNA in 10 mM phosphate buffer was determined to be 69 °C. When the measurements were repeated at a Bp/D of 5 in 10 mM phosphate buffer, complexes **Ru-C_3_-Nap-4NO_2_**, **Ru-C_3_-2Nap-4NO_2_**, and **Ru-C_5_-Nap-4NO_2_** were observed to increase the melting temperature to a similar degree (ca. 6–7 °C), see Figure 6. It should be noted that for these systems the melting transition was incomplete to at 90 °C and it was necessary to extrapolate the curve by fitting to a sigmoidal function. Thus, the values reported should be regarded as approximations and are likely underestimated. Interestingly, the bis-pentyl **Ru-C_5_-2Nap-4NO_2_** complex gave only a small increase in *T*_m_. A similar observation was made in our previous study of the structurally related unsubstituted naphthalimide C_5_ linker complexes **Ru-C_5_-Nap** and **Ru-C_5_-2Nap**.^44^ In that study, the mono naphthalimide was found to stabilize the *T*_m_ by 6.8 °C while the bis complex only caused a 2.0 °C increase. This observation was taken to suggest that the naphthalimide groups were loosely associated or bound in a manner that does not stabilize the double helix effectively, which may be further impacted here by the presence of the nitro substituent. A small difference in *T*_m_ was observed between **Ru-C_3_-2Nap-4NO_2_** and its mono-naphthalimide analogue **Ru-C_3_-Nap-4NO_2_**, possibly indicating that only one of the naphthalimide moieties in **Ru-C_3_-2Nap-4NO_2_** may be bound by classical intercalation. This result is also in agreement with the observations from the UV/visible absorption titration.

### Circular Dichroism (CD) Studies

**Figure 7:**
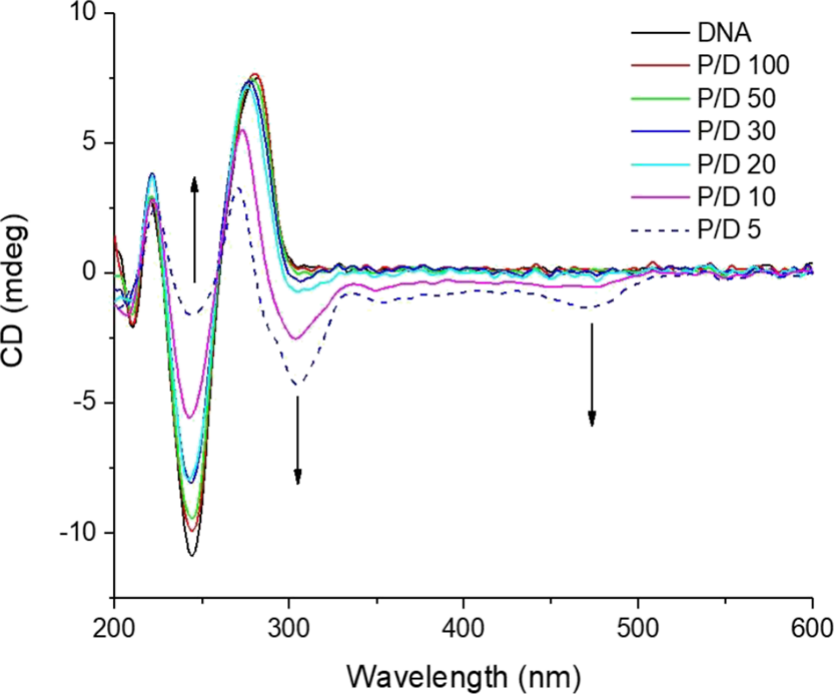
CD curves of st-DNA (150 μM) in 10 mM phosphate buffer, pH 7, in the absence and presence of **Ru-C_5_-2Nap-4NO_2_** at varying ratios.

CD is capable of reporting on the electronic coupling between chiral DNA and the bound species. The phenomenon is most readily observed in regions that do not overlap with DNA bands (greater than 300 nm). The CD spectra recoded for DNA (150 μM) in the presence of different concentrations of the racemic control complex Ru-1 revealed no induced signal arising from the complex (Figure S10). However, the spectrum recorded for **Ru-C_5_-2Nap-4NO_2_** showed a structured CD signal with contributions at 350 nm (nap) and at longer wavelength, centered at 475 nm, for the MLCT absorption, see Figure 7. This result indicates that both the naphthalimide and Ru(II)-polypyridyl complex are bound to DNA. Changes in CD are also observed in the DNA region as shown from the difference spectra. However, as spectral changes in this region could result either from a conformational change in the DNA upon binding of the complexes or from induced CD of the bound complex we do not use the changes to draw any conclusions. Interestingly, a significantly weaker signal was observed for the **Ru-C_3_-2Nap-4NO_2_**, Figure S11. It is known that the location in the groove yields a greater CD signal and the reduced signal may reflect a different binding site or could indicate that both enantiomers are binding equally to DNA.

### DNA Nicking Experiments

**Figure 8:**
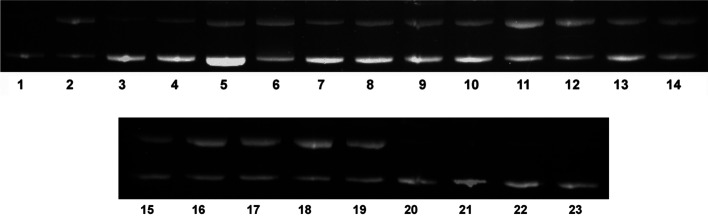
Agarose gel electrophoresis of pBR322 DNA (1 mg/mL) after 5 min irradiation at λ > 400 nm in 10 mM phosphate buffer, pH 7. Lane 1: Plasmid DNA control; Lane 2: Ru(bpy)_3_^2+^ (Bp/D 25); Lane 3: 10 mM Histidine; Lanes 4–6: **Ru-C_5_-2Nap-4NO_2_** (Bp/D 25, 15, 5); Lanes 7–9: **Ru-C_3_-2Nap-4NO_2_** (Bp/D 25, 15, 5); Lanes 10–12: **Ru-C_5_-Nap-4NO_2_** (Bp/D 25, 15, 5); Lanes 13–15: **Ru-C_3_-Nap-4NO_2_** (Bp/D 25, 15, 5); Lanes 16–19: Histidine + **Ru-C_5_-2Nap-4NO_2_, Ru-C_3_-2Nap-4NO_2_, Ru-C_5_-Nap-4NO_2_, Ru-C_3_-Nap-4NO_2_** respectively (Bp/D 25, 15, 5); Lanes 20–23: **Ru-C_5_-2Nap-4NO_2_, Ru-C_3_-2Nap-4NO_2_, Ru-C_5_-Nap, Ru-C_3_-Nap-4NO_2_** respectively (Bp/D 5) in the dark.

Previous DNA nicking experiments by us found that the mono-substituted naphthalimide complex **Ru-C_5_-Nap** led to greater DNA photocleavage than the [Ru(bpy)_3_]^2+^ control. However, only minor DNA cleavage was observed for the bis-substituted naphthalimide complex **Ru-C_5_-2Nap**, comparable to that observed for [Ru(bpy)_3_]^2+^. These observations were attributed to the mono complex having a binding site that best positioned or anchored the metal center in optimal proximity with the DNA target.^44^ Next, the ability of the **4-NO_2_** complexes to cause photocleavage of DNA was examined. These complexes were expected to damage DNA by the generation of singlet oxygen (^1^O_2_), as they were not sufficiently photo-oxidizing to undergo charge transfer reactions with the DNA.^55^ However, the presence of the 1,8-naphthalimides (that anchor the Ru(II) center tightly in place) may additionally result in improved efficiency of cleavage over systems such as Ru(bpy)_3_^2+^, as previously observed for the **Ru-C_5_-Nap** complex.^44^ The results of the cleavage experiments for this family of complexes are presented in Figure 8. Lane 1 represents intact plasmid, which was directly loaded onto the gel, the presence of mostly Form I, indicating that the stock DNA was undamaged. Lanes 20–23 represent plasmid with added complexes in the dark; the absence of cleavage confirms that the complexes do not damage DNA without irradiation. Lane 2 represents the control photocleaver Ru(bpy)_3_^2+^, which was used to evaluate the relative efficiency of cleavage of the conjugate systems. Ru(bpy)_3_^2+^ is known to be an efficient ^1^O_2_ sensitizer and to remove Form I of plasmid DNA upon irradiation with visible light.^62,63^ The observation that this process is suppressed in the presence of Mg^2+^ indicates that binding of the ruthenium complex is essential for cleavage.^63^ The efficiency of Ru(bpy)_3_^2+^ photocleavage is limited by its modest DNA binding affinity (0.7 × 10^3^ M^–1^), which is largely electrostatic in nature.^63,64^ In spite of their strong binding affinity for DNA (10^6^–10^7^ M^–1^), the 4-nitro naphthalimide complexes were found to cleave DNA with rather poor efficiency, of approximately the same order as Ru(bpy)_3_^2+^. These initial studies suggest that the poor efficiency of photocleavage may arise due to the low quantum yield of MLCT emission determined for these systems (≤0.001). This affords a small amount of populated excited state and leads to an inefficient production of ^1^O_2_, and therefore poor efficiency of cleavage of the DNA. However, it is also expected to be related to the nature of the binding. However, in contrast to the observations for the unsubstituted **Ru-C_5_-Nap** and **Ru-C_5_-2Nap** systems^44^ the low QY appears to have a leveling effect on the complexes yielding very similar behavior.

### Photophysical and DNA Binding Studies of the **Ru-C_5_-2Nap-3NO_2_** Complex

It has been suggested in the literature that 3-nitro-1,8-naphthalimides are more effective intercalators than their 4-nitro counterparts, as in the latter, the nitro group may be twisted slightly out of plane due to steric repulsion by the neighboring H-atom in the 5-position of the ring system.^65^ Given the strong off–on light switch effect (11-fold enhancement) and induced CD signal observed for **Ru-C_5_-2Nap-4NO_2_**, this complex was chosen as a candidate to probe the influence of the nitro position on DNA binding by studying the related **Ru-C_5_-2Nap-3NO_2_** complex. In comparison to **Ru-C_5_-2Nap-4NO_2_**, the maximum of the 1,8-naphthalimide absorption band of **Ru-C_5_-2Nap-3NO_2_** was shifted to a slightly shorter wavelength of 338 nm, with ε = 13,400 M^–1^ cm^–1^, which is less than that observed for the 4-nitro equivalent. This is attributed to greater intramolecular stacking of the 3-nitro-1,8-naphthalimides, and therefore, a greater hypochromicity in absorption is observed. Excitation at 350 nm resulted in a reasonably intense naphthalimide emission at 456 nm, which also resulted in Ru(II) MCLT-based emission, see Figure S12. Again, direct excitation of the MLCT band at 450 nm resulted in low emission with a low quantum yield of <0.001, which is attributed to quenching of the metal center by electron transfer to the 4-nitro-1,8-naphthalimide moieties.

Next the binding of **Ru-C_5_-2Nap-3NO_2_** to DNA was investigated. The absorbance of the 1,8-naphthalimide band at 338 nm in **Ru-C_5_-2Nap-3NO_2_** was found to increase (10%) upon the initial addition of st-DNA, up to 0.6 base pair equivalents, which is attributed to loss of hypochromism due to a disruption of intramolecular stacking of the naphthalimide groups (Figure S13). After this, the subsequent addition of DNA resulted in up to ca. 30% hypochromism (relative to the starting point), which is likely due to insertion of the 1,8-naphthalimides into the DNA helix. In contrast to the **Ru-C_5_-2Nap-4NO_2_** complex, the addition of DNA also resulted in a significant hypochromism (18%) in the MLCT band and suggests greater interaction of the polypyridyl complex. Direct comparison of the change in the absorbance of the naphthalimide band at 350 nm for the 3-nitro and 4-nitro complexes clearly shows the enhanced response of the **Ru-C_5_-2Nap-3NO_2_** complex, which is achieved with less equivalents of DNA (Figure S14).

**Figure 9:**
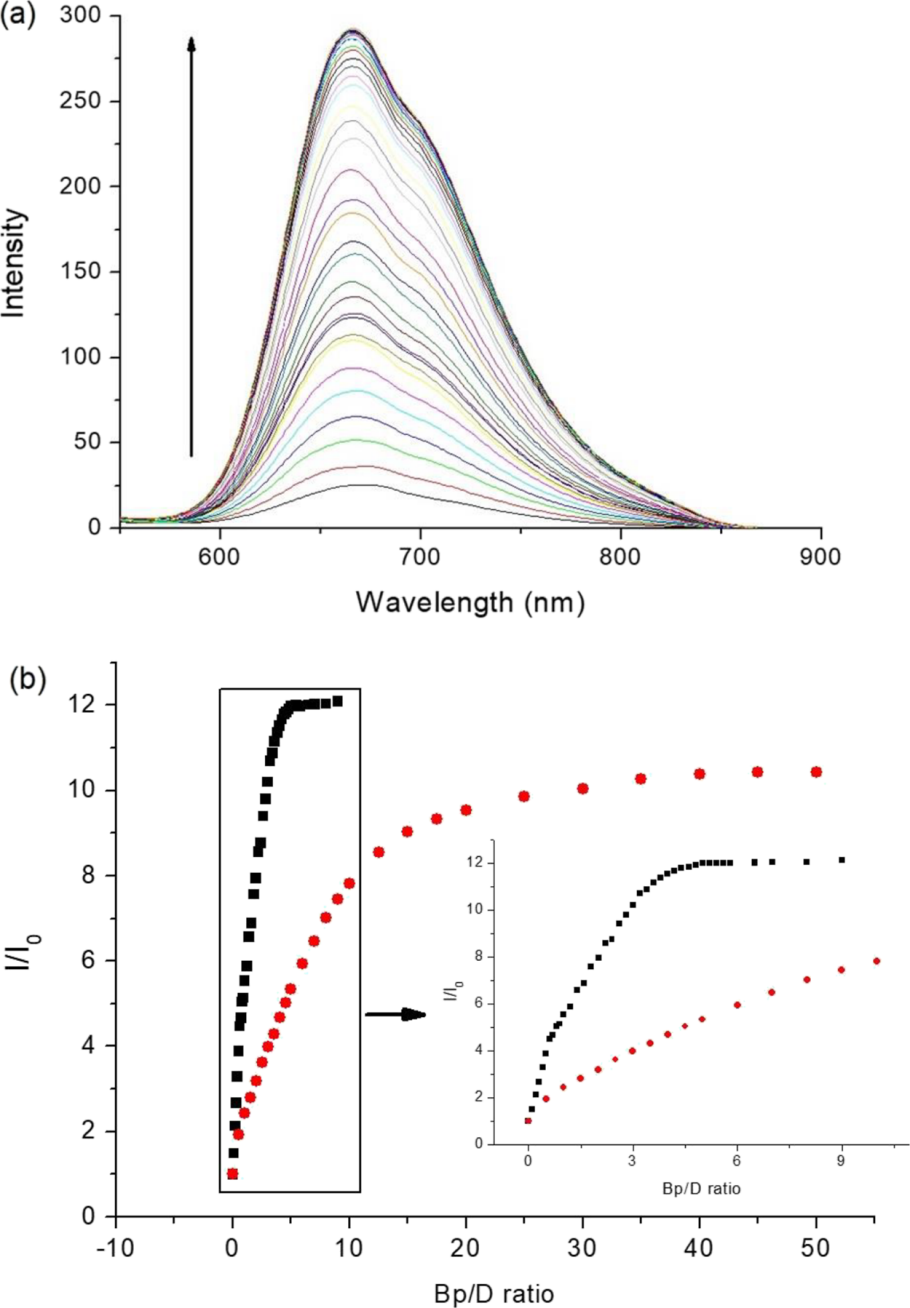
(a) Changes in the MLCT emission of 6.5 μM **Ru-C_5_-2Nap-3NO_2_** (λ_ex_ 450 nm) in the presence of st-DNA (0–58.5 μM Bp = base pairs). (b) Comparison of the emission intensity changes for 6.5 μM **Ru-C_5_-2Nap-4NO_2_** (solid circle) and **Ru-C_5_-2Nap-3NO_2_** (solid box) upon addition of st-DNA (0–58.5 μM base pairs) all in 10 mM phosphate buffer, pH 7 (λ_ex_ 450 nm).

As observed for the other complexes, the MLCT emission of **Ru-C_5_-2Nap-3NO_2_** was found to increase in the presence of DNA, see Figure 9a. Notably, the half point of the intensity change occurs at a Bp/D ratio of 1.3. A plot of the integrated intensity as a function of added DNA shows two distinct regions, see Figure 9b, which mirror the change in the profile observed for the absorption data. The steeper increase observed up to a Bp/D ratio of 0.6 is assigned to the first stage in DNA binding, in which the intermolecular stacking of the 1,8-naphthalimides is disrupted. Further addition of DNA results in a twelve-fold increase in emission with the plateau in the emission intensity reached at a significantly lower Bp/D ratio of approximately 5 compared to **Ru-C_5_-2Nap-3NO_2_** (Bp/D approx. 20). The off–on light-switch behavior of the complex was also observed under conditions of greater ionic strength where again significant enhancement was observed by 0.6 Bp/D equivalents with a reduced overall effect for 50 NaCl (10-fold) and 100 mM NaCl (6-fold), see Figure S15. The observed enhancement at lower Bp/D ratio, combined with the performance under conditions of higher ionic strength indicates a greater binding affinity for DNA, which is attributed to the 3-nitro-1,8-naphthalimide being more deeply inserted into the helix. Additionally, the quantum yield of **Ru-C_5_-2Nap-3NO_2_** was determined to be 0.012 when bound to DNA, which is quite close to that of the reference complex **Ru-1** (0.014), this would be expected for a complex where improved intercalation disrupted the quencher interaction with the metal complex. Thermal denaturation studies were also carried out on **Ru-C_5_-2Nap-3NO_2_**, which was found to stabilize duplex DNA to a greater extent than that observed for the 4-nitro-1,8- **Ru-C_5_-2Nap-4NO_2_**, giving an increase in *T*_m_ of 6 °C at a Bp/D ratio of 5, see Figure S16.

**Figure 10:**
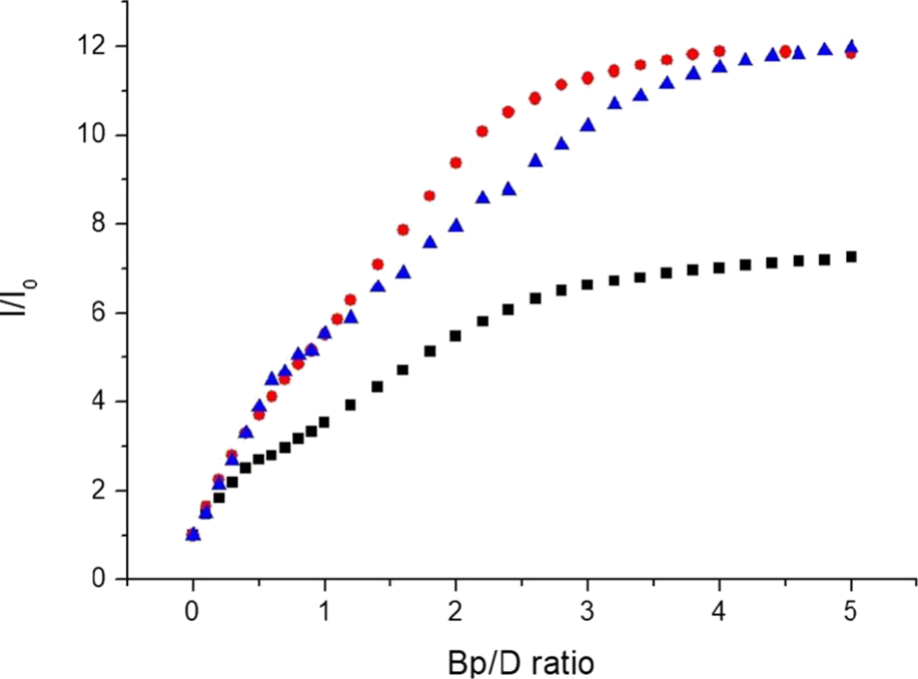
Comparison of the changes in the integrated intensity of the MCLT band upon addition of (solid upward pointing triangle) st-DNA, (solid circle) [poly(dG-dC)]_2_, and (solid box) [poly(dA-dT)]_2_ all in 10 mM phosphate buffer, pH 7.

As the most promising chromophore-quencher probe candidate, the sensitivity of the off–on light-switch response of **Ru-C_5_-2Nap-3NO_2_** to AT and GC binding sites was investigated by performing titrations with the [poly(dG-dC)]_2_ and [poly(dA-dT)]_2_ homopolymers, see Figures 10 and S17 and S18. The emission profile observed for **Ru-C_5_-2Nap-3NO_2_** in the presence of increasing concentrations of [poly(dG-dC)]_2_ is found to be very similar to that observed for st-DNA, with again a two-phase process visible, see Figure 10 and Figure S17. However, the second part of the profile is slightly steeper than that seen in the presence of st-DNA. Consequently, the plateau in emission intensity is reached at slightly lower base pair equivalents with the same overall enhancement. Notably, the emission enhancement observed in the presence of [poly(dA-dT)]_2_ followed the same basic profile of enhancement, but the magnitude of the overall change was less at 7.3 fold, see Figures 10 and S18.

Overall, the changes in the emission of **Ru-C_5_-2Nap-3NO_2_** upon the addition of st-DNA, [poly(dG-dC)]_2_ and [poly(dA-dT)]_2_ follow the same shape profile and so may be regarded as possessing similar affinity for the different DNA sequences. The difference in the luminescence response to the homopolymers is likely due to the difference in the structure of AT and GC sequences, where for instance the minor groove in AT rich regions is narrower,^66^ and AT base pairs have a greater propensity to adopt twisted propeller conformations.^67^ These regions likely give rise to different binding modes that influence the emission response. A related phenomenon has previously been observed for the [Ru(phen)_2_dppz]^2+^ light-switch complex.^68^ In this case, the greater emission observed for [poly(dA-dT)]_2_ compared to [poly(dG-dC)]_2_ was attributed to the different binding sites available to the complex. In this study, the narrower minor groove in [poly(dA-dT)]_2_ likely causes the **Ru-C_5_-2Nap-3NO_2_** to adopt a different binding mode, which is less disruptive of the quenching mechanism. It is notable that binding of **Ru-C_5_-2Nap-4NO_2_** to st-DNA and [poly(dG-dC)]_2_ leads to a very similar luminescence enhancement, even though the former possesses a heterogeneous base content. The similarity between the results may reflect their similar overall structure of st-DNA and [poly(dG-dC)]_2_ or indicate a preference for binding at GC sites. Overall, these results demonstrate the impact of modification of the naphthalimide intercalator on the DNA binding ability and the chromophore-quencher probe capability.

## Conclusions

A new family of bifunctional Ru(II) polypyridyl complexes possessing either a single or two 4-nitro-1,8-naphthalmide subunits were synthesized where the two chromophoric moieties are separated by aliphatic spacers of either 3 or 5 carbons. The complexes are found to be weakly emissive in aqueous solution, exhibiting naphthalimide or MLCT-based emission depending on the excitation wavelength. In all cases, a low QY of MLCT emission is observed in aqueous solution at pH 7, and this is attributed to intramolecular electron transfer quenching by the appended naphthalimides. The complexes were found to bind DNA with high binding constants. In the presence of DNA, the Ru(II)-4-nitro naphthalimide systems displayed significant emission enhancement. The ability of the complexes to behave as chromophore quencher DNA probes was found to depend on both the length of the linker and the number of naphthalimide groups. It was shown that the pentyl linkers **Ru-C_5_-Nap-4NO_2_** and **Ru-C_5_-2Nap-4NO_2_** gave rise to greater emission enhancement than the corresponding propyl analogues **Ru-C_3_-Nap-4NO_2_** and **Ru-C_3_-2Nap-4NO_2_**, which is most likely due to greater separation of the chromophore and quencher upon DNA binding. Of these systems, the bis-pentyl **Ru-C_5_-2Nap-4NO_2_** performed best as an off–on light-switch with an 11-fold enhancement in the luminescence. 1,8-Naphthalimides are known to be effective DNA intercalators^37,69^ and while a definitive assignment of binding is not possible in the absence of viscosity measurements, the combination of the strong binding constants, stabilization to denaturation, and spectroscopic changes observed strongly suggest a role for intercalation in these systems. The study further demonstrates the versatility of naphthalimides as sensitive probes of their environment. Here, a slight structural modification of the 1,8-naphthalimide, involving a change in the substitution pattern of the nitro group, results in a significant effect on the binding and emission properties of the resulting complex. In this way, appropriate choice of the naphthalimide DNA binding “hook” with the appropriate linker “line” allows improved capture of DNA; this design results in a highly effective luminescent reporting structure, the “sinker”! A quite novel feature of these complexes is that the more diagnostically favorable metal complex emission effectively reports on the change in the environment experienced by the naphthalimide moieties, which emit at significantly lower wavelengths. In conclusion, this work further highlights the versatility and potential of ruthenium(II) conjugates as powerful DNA probes.

## Experimental Section

### General Techniques

^1^H NMR spectra were recorded at 400 MHz using a Bruker Spectrospin DPX-400 instrument. ^13^C NMR spectra were recorded at 100 MHz using a Bruker Spectrospin DPX-400 instrument. Mass spectrometry was carried out using HPLC grade solvents. Mass spectra were determined by detection using electrospray on a Micromass LCT spectrometer. High-resolution mass spectra were determined by a peak matching method, using leucine Enkephalin, (Tyr-Gly-Gly-Phe-Leu), as the standard reference. Melting points were determined using an IA9000 digital melting point apparatus. Infrared spectra were recorded on a PerkinElmer spectrometer fitted with a Universal ATR Sampling Accessory. Elemental analysis was conducted in the Microanalytical Laboratory, School of Chemistry, University College Dublin. UV–visible absorption and UV thermal denaturation experiments spectra were recorded on a thermoelectrically coupled Varian Cary 50 spectrometer. The temperature in the cell was ramped from 20 to 90 °C, at a rate of 1 °C min^–1^ and the absorbance at 260 nm was measured every 0.2 °C. CD spectra were recorded at a concentration corresponding to an optical density of approximately 1.0 in buffered solutions on a Jasco J-810-150S spectropolarimeter. Emission spectra were recorded on a Cary Eclipse Luminescence spectrometer. The luminescence quantum yields (Φ_f_) were calculated by comparison with [Ru(bpy)_3_]^2+^ in aerated aqueous solutions prepared at a number of concentrations all with absorbance less than 0.1 at room temperature. Emission spectra were recorded for each sample, and the integrated intensity was obtained. Plots of integrated intensity vs absorbance were constructed and the slope of the line through the points determined. This gradient was substituted into eq 1 from which the QY was determined where the subscripts ST and X denote the standard and sample, respectively, Φ is the fluorescence quantum yield, Grad is the gradient from the plot of integrated fluorescence intensity vs absorbance, and η is the refractive index of the solvent.
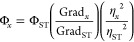
1

#### Agarose Gel Electrophoresis

The DNA photocleavage studies were carried out under aerated conditions by treating pBR322 plasmid DNA (1 mg/mL) with each of the complexes at different ratios (Bp/D of 25, 15, 5) in 10 mM phosphate buffer, pH 7. The samples were then subjected to 2 J cm^–2^ using a Hamamatsu L2570 200 W HgXe arc lamp equipped with a NaNO_2_ filter before being separated using 0.8% agarose gel electrophoresis in a TBE (90 mM Tris-borate, 2 mM EDTA, pH 8.0) buffer. Electrophoresis was carried out at 5 V/cm (40 mA, 90 V). Visualization of the DNA was achieved by staining the gel for 90 min with an aqueous solution of ethidium bromide, which was then illuminated with a transilluminator (Bioblock 254 UV illuminator).

### Materials

*cis*-[Ru(bpy)_2_Cl_2_].2H_2_O,^70^ 4,4′-dicarboxy-2,2′-bipyridine,^71^ and 4-carboxy-4′-methyl-2,2′-bipyridine^72^ were prepared by the literature routes. All other reagents and solvents were purchased commercially and used without further purification. Solutions of salmon testes DNA (st-DNA) in 10 mM phosphate buffer (pH 7) gave a ratio of UV absorbance at 260 and 280 nm of 1.86:1, indicating that the DNA was sufficiently free of protein. Its concentration was determined spectrophotometrically using the molar absorptivity of 6600 M^–1^ cm^–1^ (260 nm). [poly(dA-dT)]_2_ and [poly(dG-dC)]_2_ were purchased from Amersham Biosciences, and their concentration was determined spectrophotometrically using the molar absorptivities of 6100 M^–1^ cm^–1^ (262 nm) and 8400 M^–1^ cm^–1^ (254 nm), respectively. Binding constants were determined by absorption titration of the complexes with DNA at room temperature, in 10 mM phosphate buffer at pH 7. Fits of experimental data were performed with Origin software.

### Synthesis of **Ru-1**

A solution of 4,4′-bis(propylcarboxamide)-2,2′-bipyridine (0.12 g, 0.34 mmol, 1.1 equiv) and Ru(bpy)_2_Cl_2_.2H_2_O (0.16 g, 0.31 mmol, 1 equiv) in DMF/H_2_O was degassed by bubbling with argon for 10 min followed by reflux under an argon atmosphere for 24 h. The solvent was removed under reduced pressure, and the residue was dissolved in H_2_O and filtered. The filtrate was reduced in volume and to it was added a concentrated aqueous solution of NH_4_PF_6_. The resulting precipitate was extracted with CH_2_Cl_2_ and dried over MgSO_4_, and the solvent was removed under reduced pressure. After purification by silica flash column chromatography, eluting with CH_3_CN/H_2_O/NaNO_3(sat)_ 40:4:1 is performed. To remove excess NaNO_3_, the solvent was removed under reduced pressure and the resulting solid was stirred vigorously in the CH_3_CN for 2 h. The resulting suspension was filtered through a double thickness of filter paper, the supernatant was isolated, and the solvent was removed under reduced pressure. The nitrate salt of the complex was then dissolved in water and the PF_6_^–^ complex was reformed and precipitated from water by dropwise addition of conc. aq. NH_4_PF_6_. The precipitate was washed with H_2_O (5 mL × 2) via centrifugation as above and was dried under vacuum. The chloride form of the complex was reformed by swirling a solution of the PF_6_^–^ complex in MeOH (15 mL) in Amberlite ion exchange resin (chloride form) for 1 h, filtered, and dried under vacuum. The product was obtained as a red/brown solid (0.17 g, 70%). Found C 43.61%, H 3.85%, N 9.86%. C_40_H_42_F_12_N_8_O_2_P_2_Ru.0.66CH_2_Cl_2_ requires C 43.83%, H 3.92%, N 10.05%; ^1^H NMR (CD_3_CN, 400 MHz) δ 8.94 (2H, s, Bpy-H), 8.53 (4H, d, *J* = 7.5 Hz), 8.09 (4H, dd, *J* = 13.1, 7.0 Hz), 7.89 (2H, d, *J* = 6.0 Hz, Bpy-H), 7.72 (6H, m, Bpy-H), 7.53 (2H, br s, NH), 7.42 (4H, m, Bpy-H), 3.42 (4H, q, *J* = 6.5 Hz, CH_2_), 1.61 (4H, m, CH_2_), 1.41 (4H, h, *J* = 7.5 Hz, CH_2_), 0.96 (6H, t, *J* = 7.5 Hz, CH_3_); ^13^C NMR (CD_3_CN, 100 MHz) δ 163.9, 158.4, 157.8, 157.7, 153.4, 152.7, 152.5, 143.7, 139.0, 128.64, 128.58, 125.8, 125.3, 122.9, 40.5, 32.0, 20.7, 14.0; ESI-MS *m*/*z* 384.1220 (M)^+^; IR (cm^–1^) 1644 (s, −CONH−).

### Synthesis of **Ru-C_3_-Nap-4NO_2_**

#### Ru-C_3_-Nap-4NO_2_

**Ru-C_3_-Nap-4NO_2_** was synthesized according to the same procedure as **Ru-1** using 4-[*N*-(propylcarboxamide)-4-nitro-1,8-naphthalimide]-4′-methyl-2,2′-bipyridine (0.20 g, 0.41 mmol, 1 equiv) and Ru(bpy)_2_Cl_2_.2H_2_O (0.24 g, 0.45 mmol, 1.1 equiv) giving the product as a red/brown solid (0.240 g, 60%). Found C 46.12%, H 3.02%, N 9.90%. C_47_H_37_F_12_N_9_O_5_P_2_Ru·CH_3_OH requires C 46.39%, H 3.23%, N 10.36%. ^1^H NMR (CD_3_CN, 600 MHz) δ 8.79 (1H, s, NH), 8.66 (1H, d, *J* = 8.6 Hz, Ar-H), 8.58 (1H, d, *J* = 7.1 Hz, Ar-H), 8.55 (1H, d, *J* = 8.1 Hz, Ar-H), 8.51 (5H, m, 5 × Ar-H), 8.36 (1H, d, *J* = 8.0 Hz, Ar-H), 8.05 (4H, m, 4 × Ar-H), 7.94 (1H, m, Ar-H), 7.86 (1H, d, *J* = 5.9 Hz, Ar-H), 7.73 (5H, m, 5 × Ar-H), 7.63 (1H, d, *J* = 5.9 Hz, Ar-H), 7.56 (1H, d, *J* = 5.7 Hz, Ar-H), 7.40 (4H, m, 4 × Ar-H), 7.27 (1H, d, *J* = 5.7 Hz, Ar-H), 4.17 (2H, t, *J* = 7.2 Hz, CH_2_), 3.48 (2H, m, CH_2_), 2.54 (3H, s, CH_3_), 2.02 (2H, m, CH_2_); ^13^C NMR (CD_3_OD, 150 MHz) δ 164.1, 163.1, 162.2, 157.9, 157.0, 156.9, 156.8, 156.0, 151.9, 151.2, 151.0, 150.8, 150.3, 149.2, 142.2, 137.9, 137.8, 137.7, 131.5, 129.4, 129.3, 128.7, 128.5, 128.3, 127.6, 127.4, 126.2, 125.6, 124.8, 124.3, 124.2, 123.6, 122.9, 122.4, 121.5, 37.9, 37.5, 27.2, 19.9, ESI-MS *m*/*z* 909.1982 (M)^2+^; IR (cm^–1^) 1704 (w, −CO–N–CO−), 1659 (m, −CONH−), 1525 (m, C–NO_2_), 1342 (m, C–NO_2_).

### Synthesis of **Ru-C_5_-Nap-4NO_2_**

#### Ru-C_5_-Nap-4NO_2_

**Ru-C_5_-Nap-4NO_2_** was synthesized according to the same procedure as **Ru-1** using 4-[*N*-(pentylcarboxamide)-4-nitro-1,8-naphthalimide]-4′-methyl-2,2′-bipyridine (0.38 g, 0.72 mmol, 1 equiv) and Ru(bpy)_2_Cl_2_.2H_2_O (0.37 g, 0.72 mmol, 1 equiv) giving the product as a red/brown solid (0.56 g, 65%). Found C 47.57%, H 3.36%, N 9.98%. C_50_H_44_F_12_N_9_O_5_P_2_Ru requires C 47.53%, H 3.24%, N 10.39%; ^1^H NMR (CD_3_CN, 400 MHz) δ 9.08 (1H, s, NH), 8.66 (3H, m, Ar-H), 8.56 (6H, m, Ar-H), 8.35 (1H, d, *J* = 8.2 Hz, Ar-H), 8.08 (4H, m, Ar-H), 7.93 (1H, dd, *J* = 8.8, 7.6 Hz, Ar-H), 7.82 (1H, d, *J* = 5.8 Hz, Ar-H), 7.75 (4H, m, Ar-H), 7.68 (1H, d, *J* = 5.2 Hz, Ar-H), 7.58 (1H, d, *J* = 5.8 Hz, Ar-H), 7.42 (4H, m, Ar-H), 7.28 (1H, d, *J* = 5.8 Hz, Ar-H), 4.09 (2H, m, CH_2_), 3.41 (2H, dd, *J* = 12.3, 6.4 Hz, CH_2_), 2.54 (3H, s, CH_3_), 1.71 (4H, m, 2CH_2_), 1.46 (2H, m, CH_2_); ^13^C NMR (CD_3_CN, 100 MHz) δ 162.9, 162.6, 162.1, 157.4, 156.6, 156.5, 156.4, 155.8, 151.7, 151.3, 151.2, 151.1, 150.3, 150.2, 150.0, 142.3, 137.4, 137.3, 131.2, 129.4, 128.9, 128.4, 128.3, 128.1, 127.1, 126.8, 125.3, 124.5, 123.9, 123.8, 123.6, 122.8, 121.0, 39.8, 39.2, 28.2, 26.8, 23.6, 19.8; ESI-MS *m*/*z* 937.2228 (M)^+^; IR (cm^–1^) 1703 (m, −CO–N–CO−), 1657 (s, −CONH−), 1526 (m, C–NO_2_), 1340 (m, C–NO_2_).

### Ru-C_3_-2Nap-4NO_2_

**Ru-C_3_-2Nap-4NO_2_** was synthesized according to the same procedure as **Ru-1** using 4,4′-bis-([*N*-propylcarboxamide]-4-nitro-1,8-naphthalimide)-2,2′-bipyridine (0.080 g, 0.099 mmol, 1.1 equiv) and Ru(bpy)_2_Cl_2_.2H_2_O (0.047 g, 0.090 mmol, 1 equiv) giving the product as a red/brown solid (0.060 g, 52%). Found C 54.00%, H 3.82%, N 12.92%. C_62_H_46_Cl_2_N_12_O_14_Ru·H_2_O·CH_3_CN·CH_2_Cl_2_ requires C 54.40%, H 3.72%, N 12.69%; ^1^H NMR (CD_3_CN, 400 MHz) δ 9.25 (2H, s, Ar-H), 8.85 (2H, m, NH), 8.56 (4H, m, Ar-H), 8.51 (2H, d, *J* = 8.5 Hz, Ar-H), 8.39 (4H, m, Ar-H), 8.23 (2H, d, *J* = 8.0 Hz, Ar-H), 8.11 (6H, m, Ar-H), 7.90 (2H, d, *J* = 6.0 Hz, Ar-H), 7.82–7.76 (10H, m, Ar-H), 7.46 (6H, m, Ar-H), 4.20 (4H, m, CH_2_), 3.52 (4H, m, CH_2_), 2.01 (4H, m, CH_2_); ^13^C NMR (CD_3_CN, 100 MHz) 162.3, 157.4, 156.8, 156.7, 152.2, 151.8, 151.5, 149.2, 142.5, 138.0, 137.9, 131.6, 129.6, 129.3, 128.7, 128.4, 127.6, 127.5, 126.7, 125.5, 124.2, 124.1, 123.9, 122.9, 122.7, 121.7, 38.3, 37.8, 27.5; ESI-MS *m*/*z* 1220.2505 (M)^+^; IR (cm^–1^) 1704 (m, −CO–N–CO−), 1659 (m, −CONH−), 1527 (s, C–NO_2_), 1332 (s, C–NO_2_).

#### Ru-C_5_-2Nap-4NO_2_

**Ru-C_5_-2Nap-4NO_2_** was synthesized according to the same procedure as used to prepare **Ru-1** using 4,4′-bis-[(*N*-pentylcarboxamide)-4-nitro-1,8-naphthalimide]-2,2′-bipyridine (0.078 g, 0.009 mmol, 1 equiv) and Ru(bpy)_2_Cl_2_.2H_2_O (0.049 g, 0.0095 mmol, 1.05 equiv) giving the product as a red/brown solid (0.06 g, 49%). Found 52.49%, H 4.02%, N 10.95%. C_66_H_54_Cl_2_N_12_O_14_Ru.2.5CH_2_Cl_2_ requires C 52.76%, H 3.81%, N 10.78%; ^1^H NMR (CD_3_OD, 400 MHz) δ 9.46 (2H, s, NH), 8.78 (4H, m, Ar-H), 8.38 (2H, m, Ar-H), 8.32 (4H, m, Ar-H), 8.19 (6H, m, Ar-H), 8.04 (2H, m, Ar-H), 7.88 (6H, m, Ar-H), 7.76 (2H, m, Ar-H), 7.56 (2H, m, Ar-H), 3.97 (4H, m, CH_2_), 3.42 (4H, m, CH_2_), 1.70 (8H, m, CH_2_), 1.46 (4H, m, CH_2_); ESI-MS *m*/*z* 1276.3088 (M)^2+^; IR (cm^–1^) 1704 (w, −CO–N–CO−), 1657 (s, −CONH−), 1526 (m, C–NO_2_), 1341 (C–NO_2_).

#### Ru-C_5_-2Nap-3NO_2_

**Ru-C_5_-2Nap-3NO_2_** was synthesized dissolving **4** (0.13 g, 0.15 mmol, 1 equiv) in DMF and adding water until it began to precipitate. A few drops of DMF were added to fully dissolve the ligand and Ru(bpy)_2_Cl_2_.2H_2_O (0.08 g, 0.15 mmol, 1 equiv) was added. The solution was saturated with argon by bubbling for 10 min. The reaction mixture was heated at reflux under an argon atmosphere for 24 h. The solvent was removed under reduced pressure, and the resulting residue was re-dissolved in water and filtered. The filtrate was reduced in volume and to it was added a concentrated aqueous solution of NH_4_PF_6_. The resulting precipitate was extracted with CH_2_Cl_2_ and dried over MgSO_4_, and the solvent was removed under reduced pressure. The product was purified by silica flash column chromatography eluting with CH_3_CN/H_2_O/Aqueous NaNO_3(sat)_ 40:4:1. The chloride form of the complex was reformed by stirring a solution of the PF_6_ salt in methanol with Amberlite ion exchange resin (Cl^–^ form) for 1 h. The product was obtained as a red/brown solid (0.093 g, 46%). Accurate MS (*m*/*z*) Calculated for C_66_H_54_N_12_O_10_Ru (M^2+^): 1276.3129. Found 1276.3110; ^1^H NMR δ_H_ (CD_3_CN, 400 MHz): 9.09 (1H, d, *J* = 2.0 Hz, Ar-H), 9.00 (1H, s, Ar-H), 8.88 (1H, d, *J* = 2.0 Hz, Ar-H), 8.56 (2H, m, Ar-H), 8.53 (1H, d, *J* = 3.5 Hz, Ar-H), 8.43 (1H, d, J = 8.0 Hz, Ar-H), 8.11 (2H, m, Ar-H), 7.91 (1H, d, *J* = 6.0 Hz, Ar-H), 7.86 (1H, m, Ar-H), 7.73 (3H, m, Ar-H), 7.63 (1H, br m, NH), 7.46 (1H, m, Ar-H), 7.40 (1H, m, Ar-H), 4.09 (2H, t, *J* = 7.5 Hz, CH_2_), 3.45 (2H, m, CH_2_), 1.74 (4H, m, 2 × CH_2_), 1.54 (2H, m, CH_2_), ^13^C NMR δ_C_ (CD_3_CN, 100 MHz): 164.2, 164.0, 163.4, 158.5, 157.8, 153.2, 152.8, 152.6, 150.3, 143.9, 139.0, 132.6, 130.7, 130.3, 129.7, 129.6, 128.6, 127.8, 126.5, 125.3, 124.9, 124.0, 123.9, 122.7, 41.2, 40.7, 29.5, 28.2, 25.1; IR ν_max_ (cm^–1^): 1704 (w, −CO–N–CO−), 161 (s, −CONH−), 1538 (m, C–NO_2_), 1345 (m, C–NO_2_).

## Supporting Information Available

Synthesis of intermediates and additional spectroscopic data on the DNA titrations (PDF)
