# Supplementary material for: Hook, Line, and Sinker! Spectroscopic Studies of Bi-Modular Mono- and Bis-1,8-naphthalimide-Ru(bpy)3-conjugates as DNA “Light Switches”
Source: Inorg Chem. 2022 Jul 25;61(31):12073–86. doi: 10.1021/acs.inorgchem.2c00064 (PMC9364415; doi:10.1021/acs.inorgchem.2c00064)
Supplement: Supplementary file 1 — ic2c00064_si_001.pdf [file ic2c00064_si_001.pdf]

Supporting Information

Hook, Line and Sinker! Spectroscopic studies of bi-modular mono- and bis-1,8-naphthalimide-Ru(bpy)$_3$-conjugates as DNA 'light switches'

Gary J. Ryan,[a] Thorfinnur Gunnlaugsson,[a,c]*Susan J. Quinn[b,c]*

[a]School of Chemistry, Trinity Biomedical Sciences Institute (TBSI), Trinity College Dublin, The University of Dublin, Dublin 2, Ireland

E-mail: gunnlaut@tcd.ie; Tel: +353 896 3459

[b]School of Chemistry, University College Dublin, Dublin 4, Ireland

E-mail: susan.quinn@ucd.ie; Tel: +353 7162407

[c]Synthesis and Solid State Pharmaceutical Centre (SSPC), Ireland.

S1 Synthesis

Procedure 1: Condensation reaction with 1,8-naphthalic anhydrides

To a mixture of the relevant amine (1.1 eq.) and 1,8-naphthalic anhydride (1 eq.) in anhydrous toluene was added triethylamine (3 eq.). The reaction mixture was heated at reflux under an argon atmosphere for 24 hours. The mixture was filtered while hot through celite, and the solvent removed under reduced pressure. Chloroform was added to the residue and the solution washed twice with 0.1 M HCl and once with water. The organic layer was dried over $MgSO_4$, filtered and the solvent removed under reduced pressure. Purification of the product by recrystallisation was carried out where necessary, details of which are provided later.

Procedure 2: Deprotection of Boc protecting group using trifluoroacetic acid

A solution of the Boc protected compound in trifluoroacetic acid (TFA) CH_2Cl_2 (1:1 v/v) was stirred at room temperature for 1 hour. The solvent was removed under reduced pressure and co-evaporated several times with CH_2Cl_2 to remove traces of TFA. The resulting product was dried under high vacuum. Further purification was not necessary.

Procedure 3: Coupling of 1,8-naphthalimide to bipyridine

The relevant 1,8-naphthalimide (1.1 or 2.1 eq.) and Et_3N (3 or 5 eq.) were dissolved in dry CH_2Cl_2, and 4,4'-bis(carbonylchloride)-2,2'-bipyridine (**2**) or 4-(carbonylchloride)-4'-methyl-2,2'-bipyridine (1 eq.), dissolved in dry CH_2Cl_2 was added dropwise. The resulting suspension was stirred at room temperature for 16 hours. The solvent was removed under reduced pressure. The resulting residue was stirred with 0.1 M HCl, filtered and washed with water. Purification was carried out as described for each compound.

_N_-(_tert_-Butoxycarbonyl)-1,3-diaminopropane

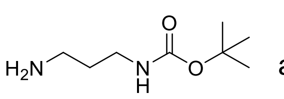

N-(*tert*-Butoxycarbonyl)-1,3-diaminopropane was synthesised by dropwise addition of a solution of Boc_2O (0.75 g, 3.45 mol, 1 eq.) in $CHCl_3$ to a solution of 1,3-diaminopropane (1.28 g, 1.44 ml, 5 eq.) in $CHCl_3$ cooled to 0 °C. The reaction mixture was stirred at room temperature overnight. The solvent was removed under reduced pressure and the residue dissolved in water and filtered. The product was extracted into CH_2Cl_2, which was then dried over $MgSO_4$, filtered and the solvent removed under reduced pressure. This yielded the product as a yellow oil (0.48 g, 80%). ^1H NMR δ_H ($CDCl_3$, 400 MHz): 4.94 (1H, br, s, N$\underline{H}$), 3.22 (2H, m, C$\underline{H}_2$), 2.77 (2H, m, C$\underline{H}_2$), 1.62 (2H, m, C$\underline{H}_2$), 1.45 (12H, s, (C$\underline{H}_3$)$_3$); ^{13}C NMR δ_C ($CDCl_3$, 100 MHz): 155.8, 78.7, 38.5, 37.6, 36.8, 27.6; ESI-MS m/z 175.1 (M+H).

_N_-[(_tert_-Butoxycarbonyl)-3-aminopropyl]-4-nitro-1,8-naphthalimide

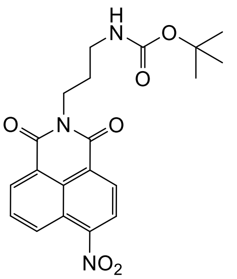

The compound was synthesized according to **Procedure 1** using 4-nitro-1,8-naphthalic anhydride (0.59 g, 2.41 mmol, 1 eq.), N-(tert-Butoxycarbonyl)-1,3-diaminopropane (0.46 g, 2.65 mmol, 1.1 eq.) and Et_3N (0.73 g, 1.01 ml, 3 eq.). After purification by recrystallisation from acetone/ether the product was obtained as a pale brown solid (0.89 g, 93%). m.p. 120-121 °C; Accurate MS (m/z) Calculated for $C_{20}H_{21}N_3NaO_4$ (M+Na): 422.1328. Found 422.1320; ^1H NMR δ_H ($CDCl_3$, 400 MHz): 8.85 (1H, d, *J* = 8.5 Hz, Naph-$\underline{H}$), 8.74 (1H, d, *J* = 7.0 Hz, Naph-$\underline{H}$), 8.70 (1H, d, *J* = 8.0 Hz, Naph-$\underline{H}$), 8.42 (1H, d, *J* = 8.0 Hz, Naph-$\underline{H}$), 8.00 (1H, m, Naph-$\underline{H}$), 5.16 (1H, br s, N$\underline{H}$), 4.28 (2H, m, C$\underline{H}_2$), 3.19 (2H, d, *J* = 5.5 Hz, C$\underline{H}_2$), 1.96 (2H, m, C$\underline{H}_2$), 1.46 (9H, s, (C$\underline{H}_3$)$_3$); ^{13}C NMR δ_C ($CDCl_3$, 400 MHz): 163.1, 162.3, 155.5, 149.2, 132.2, 129.5, 129.0, 128.6, 126.3, 123.5, 123.2, 122.3, 78.7, 37.7, 37.1, 30.0.

N-(Propylammonium)-4-nitro-1,8-naphthalimide trifluoroacetate (1)

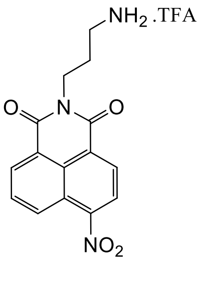

Compound **1** was synthesised according to **Procedure 2** from N-[(tert-Butoxycarbonyl)-3-aminopropyl]-4-nitro-1,8-naphthalimide (0.8 g, 2 mmol, 1 eq.) in TFA/CH_2Cl_2 (10 ml). The product was obtained as an orange oil, which later became an orange solid (0.83 g, 100%). m.p. 120 °C decomp.; ^1H NMR δ_H ([D_6]DMSO, 400 MHz): 8.67 (1H, d, *J* = 8.5 Hz, Naph-$\underline{H}$), 8.61 – 8.51 (3H, m, 3 x Naph-$\underline{H}$), 8.07 (1H, t, *J* = 8.0 Hz, Naph-$\underline{H}$), 7.85 (3H, br s, N$\underline{H}_3^+$), 4.11 (2H, m, C$\underline{H}_2$), 2.92 (2H, m, C$\underline{H}_2$), 1.98 (2H, m, C$\underline{H}_2$); ^{13}C NMR δ_C ([D_6]DMSO, 100 MHz): 163.3, 162.5, 149.1, 149.0, 131.7, 130.1, 129.6, 128.8, 128.4, 126.7, 124.2, 122.8, 122.7, 37.4, 37.0, 25.8.

4,4'-Bis-{[*N*-propylcarboxamide]-4-nitro-1,8-naphthalimide}-2,2'-bipyridine (3)

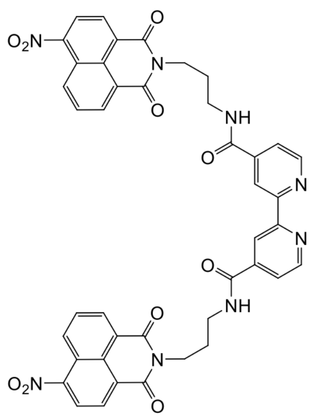

Compound **3** was synthesised according to **Procedure 3** using **1** (0.300 g, 0.73 mmol, 2.1 eq.), Et_3N (0.175 g, 0.24 ml, 1.73 mmol, 5 eq.) and 4,4'-bis(carbonylchloride)-2,2'-bipyridine (0.097 g, 0.35 mmol, 1 eq.). After purification by trituration with MeOH the product was obtained as a brown solid (0.15 g, 55%). m.p. 230 °C; Calculated for $C_{42}H_{30}N_8O_{10}.CH_3OH$: C, 61.57; H, 4.09; N, 13.36. Found: C, 61.57; H, 3.87; N, 13.39; ^{1}H NMR δ_H ([D$_6$]DMSO, 400 MHz): 9.02 (2H, t, *J* = 5.5 Hz, N$\underline{H}$), 8.81 (2H, d, *J* = 5.0 Hz, Bpy-$\underline{H}_6$), 8.71 (2H, s, Bpy-$\underline{H}_3$), 8.67 (2H, d, *J* = 9.6 Hz, Naph-$\underline{H}$), 8.59 (4H, m, 2 x Naph-$\underline{H}$), 8.52 (2H, d, *J* = 8.0 Hz, Naph-$\underline{H}$), 8.06 (2H, m, Naph-$\underline{H}$), 7.81 (2H, dd, *J* = 1.5, 5.0 Hz, Bpy-$\underline{H}_5$), 4.15 (4H, m, C$\underline{H}_2$), 3.40 (4H, m, C$\underline{H}_2$), 2.00 (4H, m, C$\underline{H}_2$); ^{13}C NMR δ_C ([D$_6$]DMSO, 100 MHz): 164.5, 163.0, 162.2, 155.4, 150.0, 149.1, 142.8, 131.7, 130.1, 129.6, 128.7, 128.4, 126.7, 124.2, 122.8, 122.7, 121.8, 118.1, 38.3, 37.5, 27.3; IR ν_{max} (cm^{-1}): 1707 (m, -CO-N-CO-), 1658 (s, -CONH-), 1526 (s, C-NO_2), 1347 (s, C-NO_2).

4-[*N*-(Propylcarboxamide)-4-nitro-1,8-naphthalimide]-4'-methyl-2,2'-bipyridine (4)

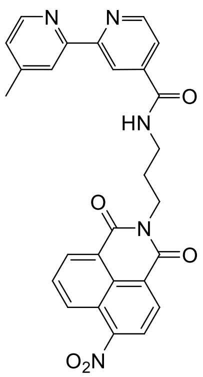

Compound **4** was synthesised according to **Procedure 3** using **1** (0.90 g, 2.18 mmol, 1.1 eq.), Et_3N (0.60 g, 0.83 ml, 5.94 mmol, 3 eq.) and 4-(carbonylchloride)-4'-methyl-2,2'-bipyridine (0.46 g, 1.98 mmol, 1 eq.). After purification by silica flash column chromatography eluting with CH_2Cl_2/MeOH 10% the product was obtained as an orange solid (0.73 g, 74%). Calculated for $C_{27}H_{21}N_5O_5.0.2CH_2Cl_2$: C, 63.75; H, 4.21; N, 13.67. Found C, 63.68; H, 4.10; N, 13.45; Accurate MS (m/z) Calculated for $C_{27}H_{22}N_5O_5$ (M+H): 496.1621. Found 496.1602; ^{1}H NMR δ_H ($CDCl_3$, 400 MHz): 8.86 (1H, d, *J* = 8.5 Hz, Ar-$\underline{H}$), 8.83 (1H, d, *J* = 5.0 Hz, Ar-$\underline{H}$), 8.80 (1H, s, Ar-$\underline{H}$), 8.78 (1H, d, *J* = 6.5 Hz, Ar-$\underline{H}$), 8.74 (1H, d, *J* = 8.0 Hz, Ar-$\underline{H}$), 8.60 (1H, d, *J* = 5.0 Hz, Ar-$\underline{H}$), 8.41 (1H, d, *J* = 8.0 Hz, Ar-$\underline{H}$), 8.30 (1H, s, Ar-$\underline{H}$), 8.03 (2H, m, 2 x Ar-$\underline{H}$), 8.00 (1H, m, Ar-$\underline{H}$), 7.83 (1H, m, Ar-$\underline{H}$), 7.53 (1H, br m, N$\underline{H}$), 7.23 (1H, d, *J* = 4.5 Hz, Ar-$\underline{H}$), 4.38 (2H, t, *J* = 6.5 Hz, C$\underline{H}_2$), 3.57 (2H, dd, *J* = 6.0, 12.5 Hz, C$\underline{H}_2$), 2.30 (3H, s, C$\underline{H}_3$), 2.16 (2H, m, C$\underline{H}_2$), ^{13}C NMR δ_C ($CDCl_3$, 100 MHz): 165.0, 163.4, 162.5, 162.1, 156.3, 154.6, 149.6, 149.3, 148.3, 142.3, 132.4, 129.8, 129.5, 129.2, 128.6, 126.1, 124.8, 123.5, 123.2, 122.2, 121.9, 121.2, 117.5, 37.8, 36.6, 27.2, 20.8,; IR ν_{max} (cm^{-1}): 1705 (w, -CO-N-CO-), 1660 (m, -CONH-), 1526 (m, C-NO_2), 1340 (m, C-NO_2).

N-(*tert*-Butoxycarbonyl)-1,5-diaminopentane (5)

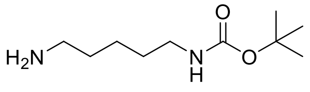

Compound **5** was synthesised by dropwise addition of Boc_2O (0.80 g, 3.67 mmol, 1 eq.) in 1,4-dioxane (20 ml) to a solution of 1,5-diaminopentane (1.50 g, 14.68 mmol, 4 eq.) in 1,4-dioxane (60 ml). The solution was stirred overnight. The solvent was removed under reduced pressure. The residue was dissolved in CH_2Cl_2 (50 ml), washed with water, dried over $MgSO_4$ and the solvent removed under reduced pressure. The product was obtained as a yellow oil (0.57 g, 82%). Accurate MS m/z Calculated for $C_{10}H_{23}N_2O_2$ (M+H): 202.1700. Found 203.1681; ^{1}H NMR δ_H ($CDCl_3$, 400 MHz): 4.63 (1H, br s, NH), 3.11 (2H, d, *J* = 6.0 Hz, CH_2), 2.68 (2H, m, CH_2), 1.53 – 1.30 (18H, m, 3 x CH_2 + $C(CH_3)_3$); ^{13}C NMR δ_C ($CDCl_3$, 100 MHz): 155.7, 78.5, 40.4, 39.8, 30.3, 29.8, 29.2, 28.0, 23.4.

N-[(*tert*-Butoxycarbonyl)-5-aminopentyl]-4-nitro-1,8-naphthalimide (6)

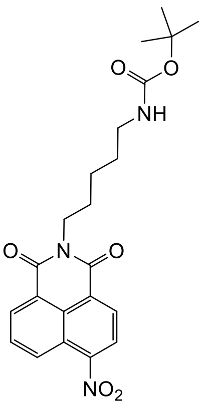

Compound **6** was synthesised according to **Procedure 1** using 4-nitro-1,8-naphthalic anhydride (0.50 g, 2.87 mmol, 1 eq.), **5** (0.60 g, 3.15 mmol, 1.1 eq.), and Et_3N (0.87 g, 1.2 ml, 8.61 mmol, 3 eq.). The product was obtained as an orange solid (1.12 g, 91%). m.p. 109 – 110 ºC; ^{1}H NMR δ_H ($CDCl_3$, 400 MHz): 8.85 (1H, d, *J* = 8.6 Hz, Naph-H), 8.74 (1H, d, *J* = 7.0 Hz, Naph-H), 8.70 (1H, d, *J* = 8.0 Hz, Naph-H), 8.42 (1H, d, *J* = 7.5 Hz, Naph-H), 8.00 (1H, m, Naph-H), 4.60 (1H, br s, NH), 4.20 (2H, t, *J* = 7.5 Hz, CH_2), 3.14 (2H, m, CH_2), 1.78 (2H, p, *J* = 7.5 Hz, CH_2), 1.60 – 1.44 (17H, m, $2CH_2$, $3CH_3$, solvent); ^{13}C NMR δ_C ($CDCl_3$, 100 MHz): 162.9, 162.0, 155.5, 149.1, 132.0, 129.5, 128.6, 127.8, 126.5, 123.5, 123.3, 122.5, 78.6, 40.1, 40.0, 29.2, 28.0, 27.2.

N-(Pentylammonium)-4-nitro-1,8-naphthalimide trifluoroacetate (7)

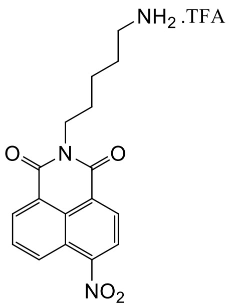

Compound **7** was synthesised according to **Procedure 2** using **6** (1.05 g, 2.46 mmol, 1 eq.). The product was obtained as an orange/brown hygroscopic solid (1.09 g, 100%). ^{1}H NMR δ_H ([D_6]DMSO, 400 MHz): 8.68 (1H, d, *J* = 8.5 Hz, Naph-H), 8.56 (3H, m, Naph-H), 8.07 (1H, dd, *J* = 8.5, 7.5 Hz, Naph-H), 7.77 (3H, br s, NH_3^+), 4.04 (2H, m, CH_2), 2.80 (2H, m, CH_2), 1.69 – 1.53 (4H, m, $2CH_2$), 1.38 (2H, m, CH_2); ^{13}C NMR δ_C ([D_6]DMSO, 100 MHz): 162.9, 162.1, 158.6, 158.2, 149.1, 131.7, 130.1, 129.6, 128.7, 128.3, 126.6, 124.2, 122.7, 51.9, 40.3, 39.2, 26.9, 26.8,

23.3; ES-MS (m/z) 328.13 $(M)^+$; IR v_{max} (cm^{-1}): 1707 (w, -CO-N-CO-), 1532 (m, C-NO_2), 1342 (m, C-NO_2).

4-[N-(pentylcarboxamide)-4-nitro-1,8-naphthalimide]-4'-methyl-2,2'-bipyridine (8)

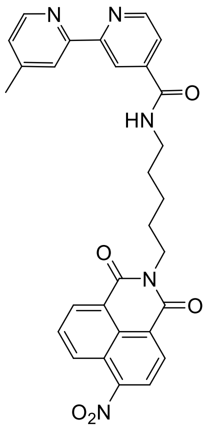

Compound **8** was synthesised according to **Procedure 3** using **7** (0.69 g, 1.56 mmol, 1.1 eq.), 4-(carbonylchloride)-4'-methyl-2,2'-bipyridine (0.33 g, 1.42 mmol, 1 eq.) and Et_3N (0.43 g, 0.60 ml, 4.26 mmol, 3 eq.). After purification by silica flash column chromatography eluting with CH_2Cl_2/MeOH 10% the product was obtained as an orange solid (0.43g, 53%). ^1H NMR δ_H ($CDCl_3$, 400 MHz): 8.79 (2H, m, 2 x Ar-H), 8.68 (2H, d, *J* = 8.6 Hz, Ar-H), 8.62 (1H, d, *J* = 8.0 Hz, Ar-H), 8.50 (1H, d, *J* = 5.0 Hz, Ar-H), 8.31 (2H, d, *J* = 8.5 Hz, Ar-H), 7.92 (1H, m, Ar-H), 7.76 (1H, dd, *J* = 5.0, 1.52 Hz, Ar-H), 7.21 (1H, d, *J* = 4.5 Hz, Ar-H), 6.84 (1H, br s, NH), 4.24 (2H, m, CH_2), 3.54 (2H, m, CH_2), 2.50 (3H, s, CH_3), 1.82 (4H, m, 2CH_2), 1.54 (2H, m, CH_2); ^{13}C NMR δ_C ($CDCl_3$, 100 MHz): 165.2, 163.0, 162.2, 154.3, 149.6, 149.0, 148.4, 148.1, 142.4, 132.0, 129.4, 129.3, 128.8, 128.7, 128.5, 126.4, 124.8, 123.4, 123.1, 122.4, 121.8, 121.6, 117.0, 39.8, 28.4, 27.1, 23.7, 20.8; IR v_{max} (cm^{-1}): 1666 (s, -CONH-), 1531 (m, C-NO_2), 1344 (m, C-NO_2)

4,4'-bis-[(*N*-pentylcarboxamide)-4-nitro-1,8-naphthalimide]-2,2'-bipyridine (9)

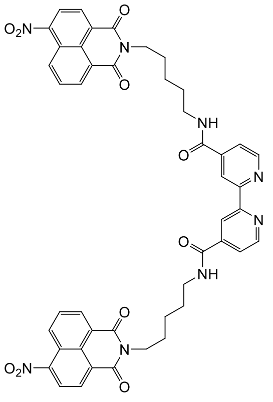

Compound **9** was synthesised according to **Procedure 3** using **7** (0.250 g, 0.565 mmol, 2.1 eq.), 4,4'-bis(carbonylchloride)-2,2'-bipyridine (0.076 g, 0.269 mmol, 1 eq.), and Et_3N (0.136 g, 0.19 ml, 1.345 mmol, 5 eq.). After purification by trituration with MeOH the product was obtained as an orange/brown solid (0.113 g, 49%). Calculated for $C_{46}H_{38}N_8O_{10}.H_20$: C, 62.72; H, 4.58; N, 12.72. Found: C, 63.05; H, 4.60; N, 13.09; ^1H NMR δ_H (DMSO[D_6], 400 MHz): 8.90 (2H, m, NH), 8.77 (2H, d, *J* = 5.0 Hz, Bpy-H_6), 8.67 (2H, s, Bpy-H_3), 8.64 (2H, d, *J* = 8.6 Hz, Naph-H), 8.56 (4H, m, Naph-H), 8.49 (2H, d, *J* = 7.5 Hz, Naph-H), 8.03 (2H, m, Naph-H), 7.74 (2H, d, *J* = 2.5 Hz, Bpy-H_5), 4.07 (2H, m, CH_2), 3.47 (2H, m, CH_2), 1.72 (2H, m, CH_2), 1.63 (2H, m, CH_2), 1.42 (2H, m, CH_2); IR v_{max} (cm^{-1}): 1706 (m, -CO-N-CO-), 1662 (s, -CONH-), 1527 (s, C-NO_2), 1342 (s, C-NO_2).

4,4'-Bis(propylcarboxamide)-2,2'-bipyridine

The compound was synthesised according to **Procedure 3** using propylamine (0.095 g, 1.30 mmol, 2 eq.), Et_3N (0.329 g, 0.45 ml, 3.25 mmol, 5 eq.) and 4,4'-bis(carbonylchloride)-2,2'-bipyridine (0.183 g, 0.65 mmol, 1 eq.). The product was obtained as a white solid (0.139 g, 60%). m.p. >250 °C decomp.;

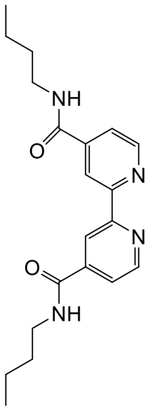

Calculated for $C_{20}H_{26}N_4O_2$: C, 67.77; H, 7.39; N, 15.81. Found: C, 67.48; H, 7.41; N, 15.64; ^{1}H NMR δ_H ([D$_6$]DMSO, 400 MHz): 8.94 (1H, m, NH), 8.87 (1H, d, *J* = 5.0 Hz, Bpy-H_6), 8.78 (1H, s, Bpy-H_3), 7.85 (1H, dd, *J* = 5.0, 1.5 Hz, Bpy-H_5), 3.31 (2H, m, CH_2), 1.55 (2H, m, CH_2), 1.35 (2H, m, CH_2), 0.92 (3H, m, CH_3); ^{13}C NMR δ_C ([D$_6$]DMSO, 100 MHz): 164.5, 155.5, 150.0, 143.1, 121.9, 118.2, 39.1, 31.0, 19.6, 13.7; IR v_{max} (cm^{-1}): 1632 (s, -CONH-).

_N_-[(_tert_-Butoxycarbonyl)-5-aminopentyl]-3-nitro-1,8-naphthalimide (11)

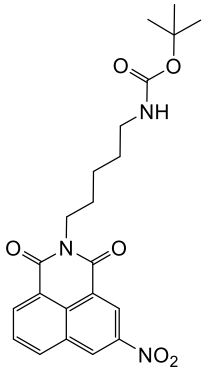

Compound **11** was synthesised according to **procedure 1** using 3-nitro-1,8-naphthalic anhydride (0.14 g, 0.81 mmol, 1 eq.), **5** (0.17 g, 0.89 mmol, 1.1 eq.), and Et_3N (0.25 g, 0.34 ml, 2.43 mmol, 3 eq.). After purification by recrystallisation from methanol the product was obtained as an orange solid (0.31 g, 90%). ^{1}H NMR δ_H ($CDCl_3$, 400 MHz): 9.27 (1H, d, *J* = 2.0 Hz, Ar-H), 9.13 (1H, d, *J* = 2.0 Hz, Ar-H), 8.77 (1H, dd, *J* = 1.0, 7.5 Hz, Ar-H), 8.44 (1H ,d, *J* = 8.0 Hz, Ar-H), 7.95 (1H, m, Ar-H), 4.64 (1H, br s, NH), 4.19 (2H, t, *J* = 7.5 Hz, CH_2), 3.14 (2H, dd, *J* = 6.5, 12.6 Hz, CH_2), 1.77 (2H, pent, *J* = 7.5 Hz, CH_2), 1.58 (2H, m, CH_2), 1.50 – 1.42 (11H, m, CH_2 + $(CH_3)_3$), ^{13}C NMR δ_C ($CDCl_3$, 100 MHz): 162.6, 162.0, 155.5, 145.9, 135.1, 134.0, 130.5, 129.7, 128.7, 128.5, 124.2, 123.7, 122.7, 78.6, 40.1, 40.0, 29.3, 28.0, 27.2, 23.8.

_N_-(pentylammonium)-3-nitro-1,8-naphthalimide trifluoroacetate (12)

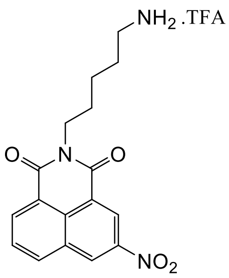

Compound **12** was synthesised according to **procedure 2** using **11** (0.26 g, 0.61 mmol, 1 eq.). The product was obtained as an orange / brown hydroscopic solid (0.26 g, 97%). Accurate MS (m/z) Calculated for $C_{17}H_{18}N_3O_4$ (M+H): 328.1297. Found 328.1295; ^{1}H NMR δ_H (CD_3OD, 400 MHz): 8.95 (1H, d, *J* = 2.0 Hz, Ar-H), 8.71 (1H, d, *J* = 2.0 Hz, Ar-H), 8.48 (1H, d, *J* = 7.0 Hz, Ar-H), 8.35 (1H, d, *J* = 8.5 Hz, Ar-H), 7.83 (1H, m, Ar-H), 4.04 (2H, m, CH_2), 3.00 (2H, m, CH_2), 1.78 (4H, m, 2 x CH_2), 1.53 (2H, m, CH_2).

4,4'-bis-[(*N*-Pentylcarboxamide)-3-nitro-1,8-naphthalimide]-2,2'-bipyridine (13)

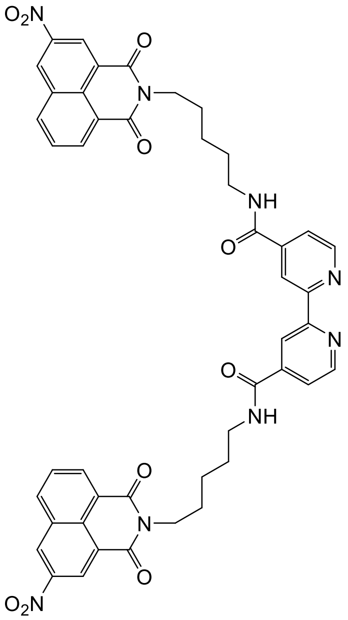

Compound **13** was synthesised according to **procedure 3** using **12** (0.20 g, 0.45 mmol, 2 eq.), 4,4'-bis(carbonylchloride)-2,2'-bipyridine (0.06 g, 0.23 mmol, 1 eq.), and Et_3N (0.12 g, 0.16 ml, 1.15 mmol, 5 eq.). The product was purified by trituration with methanol and was obtained as an orange/brown solid (0.16 g, 80%). Calculated for $C_{46}H_{38}N_8O_{10}$.1.33MeOH: C, 62.78; H, 4.82; N, 12.37. Found: C, 62.43; H, 4.54; N, 12.64; ^{1}H NMR δ_H (DMSO[D_6], 400 MHz): 9.36 (1H, d, *J* = 2.5 Hz, Ar-H), 8.89 (2H, m, Ar-H), 8.74 (1H, d, *J* = 5.0 Hz, Ar-H), 8.68 (1H, d, *J* = 8.6 Hz, Ar-H), 8.63 (2H, m, 2 x Ar-H), 7.99 (1H, m, Ar-H), 7.73 (1H, d, *J* = 5.0 Hz, Ar-H), 4.09 (2H, t, *J* = 7.0 Hz, CH_2), peak under solvent, 1.72 (2H, m, CH_2), 1.63 (2H, m, CH_2), 1.43 (2H, m, CH_2); IR ν_{max} (cm^{-1}): 1707 (w, -CO-N-CO-), 1662 (m, -CONH-), 1536 (m, C-NO_2), 1347 (m, C-NO_2).

S2 Methods: Bard Binding Model:

The intrinsic binding constant ***K*** and binding site size **n** were determined using the model derived by Bard *et al.*[29] This model is based on the equilibrium shown in equation **2** and assumes non-cooperative, non-specific binding to DNA with the existence of one discreet type of binding site. This model was chosen for fitting of the data as it takes account of all the data obtained from the titration.

The equilibrium binding constant for this process is: $K = C_b/C_fC_s$ (1)

where C_b, C_f and C_s represent the equilibrium concentrations of bound complex, free complex and free binding sites, respectively. The total concentration of complex, C_t, is: $C_t = C_b + C_f$ (2)

and the total concentration of sites along a DNA molecule with an average number of base pairs L, is

$xC_{DNA} = C_b + C_s$ (3) where $x = L/s$ (4) and $C_{DNA} = [NP]/2L$ (5)

and [NP] is the concentration of nucleotide phosphate. Solution of equations for the concentration of bound complex as a function of [NP] making appropriate substitutions yields,

$$C_b = \frac{\left(b^2 - \frac{2K^2C_t[NP]}{s}\right)^{1/2}}{2K} \quad (6)$$

where $b = 1 + KC_t + K[NP]/2s$ (7)

The expression is divided by C_t to give an expression in terms of the observed spectroscopic change,

$$(\varepsilon_a-\varepsilon_b)/(\varepsilon_b-\varepsilon_f) = \frac{\left(b^2 - \frac{2K^2C_t[NP]}{s}\right)^{1/2}}{2KC_t} \quad (8)$$

Plots of $(\varepsilon_a-\varepsilon_f)/(\varepsilon_b-\varepsilon_f)$ *vs.* [NP] were directly fitted to Eq. **8** where ε_a, ε_f and ε_b correspond to the apparent extinction coefficient at each point in the titration, the extinction coefficient for the free ruthenium complex, and the extinction coefficient for the ruthenium complex in the fully bound form respectively.

The parameters obtained represent an average which results from any number of different binding geometries.

S3 Figures

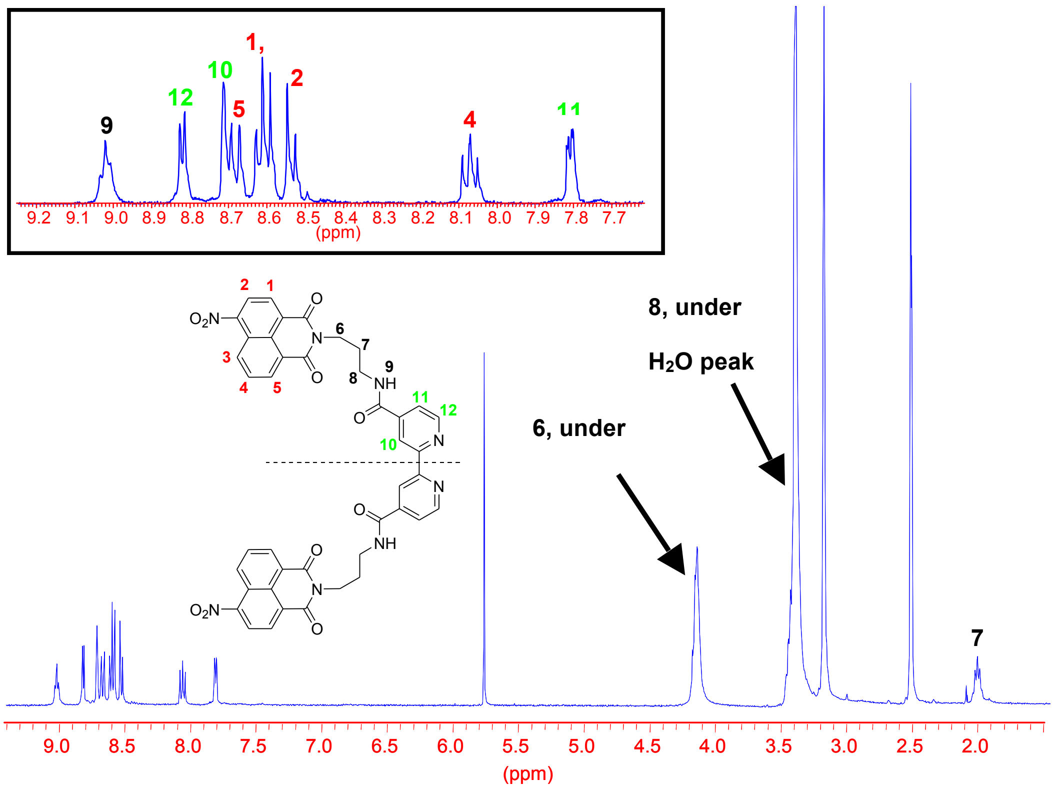

Figure S1. ^{1}H NMR spectrum of **Ru-C$_3$-2Nap-4NO$_2$** ([D$_6$]DMSO, 400 MHz).

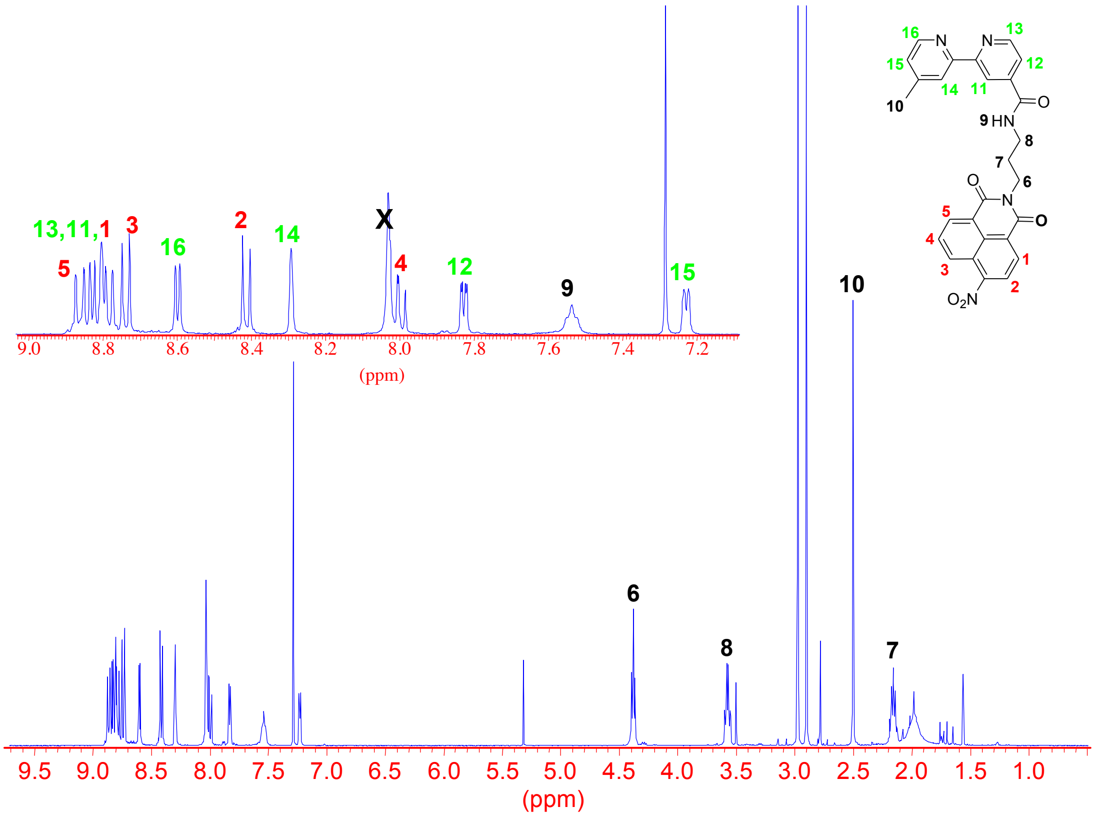

Figure S2. ^{1}H NMR spectrum of **5** ($CDCl_3$, 400 MHz).

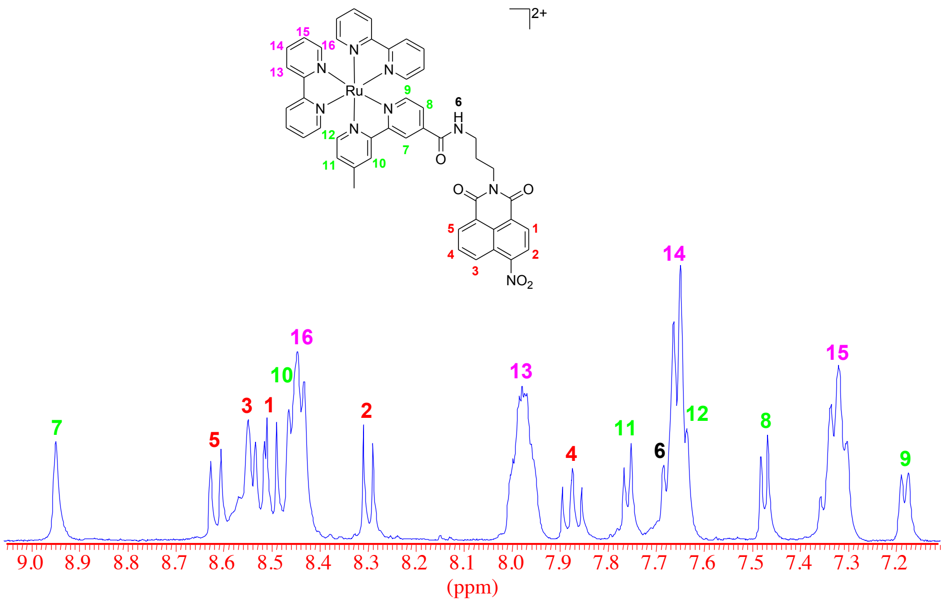

Figure S3 ^{1}H NMR spectrum of **Ru-C$_3$-Nap-4NO$_2$** (CD_3CN, 600 MHz), showing the aromatic region.

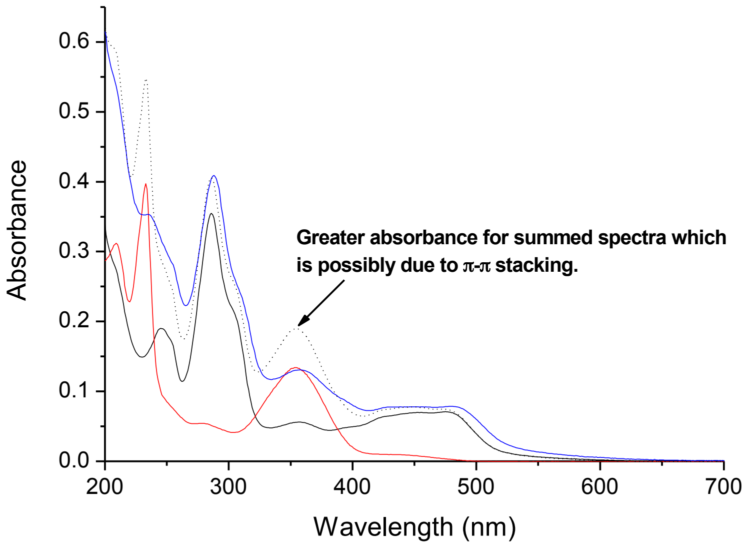

Figure S4. Summed absorption spectra for **Ru-C$_5$-2Nap-4NO$_2$**. **Ru-1** (—)(6.5 μM), **Nap-1** (—)(13 μM), **Ru-1** + **Nap-1** (------) and **Ru-C$_5$-2Nap-4NO$_2$** (——)(6.5 μM). All solutions were recorded in 10 mM phosphate buffer, at pH 7.

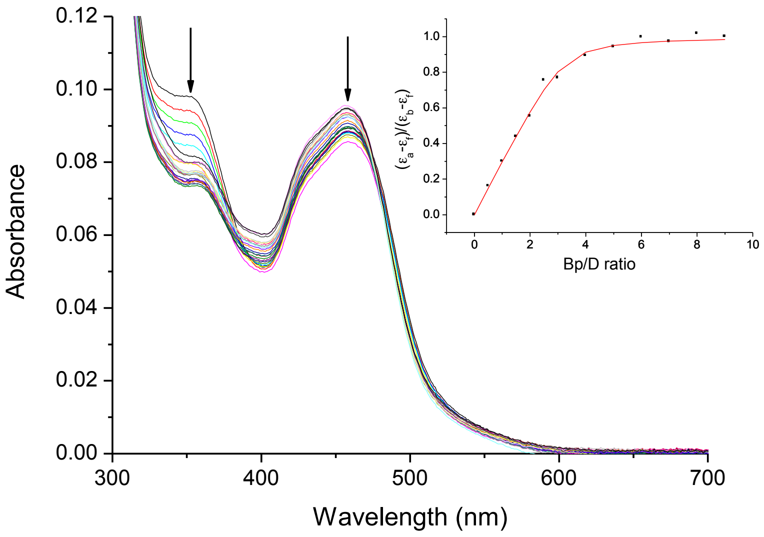

Figure S5. Changes in the UV/Visible spectrum of **Ru-C$_3$-Nap-4NO$_2$** (6.5 μM) upon addition of st-DNA (0 – 58.5 μM base pairs) in 10 mM phosphate buffer, at pH 7. Inset: Plot of $(\varepsilon_a\text{-}\varepsilon_f)/(\varepsilon_b\text{-}\varepsilon_f)$ vs. [DNA] and the corresponding non-linear fit. (Bp/D = base pair to complex).

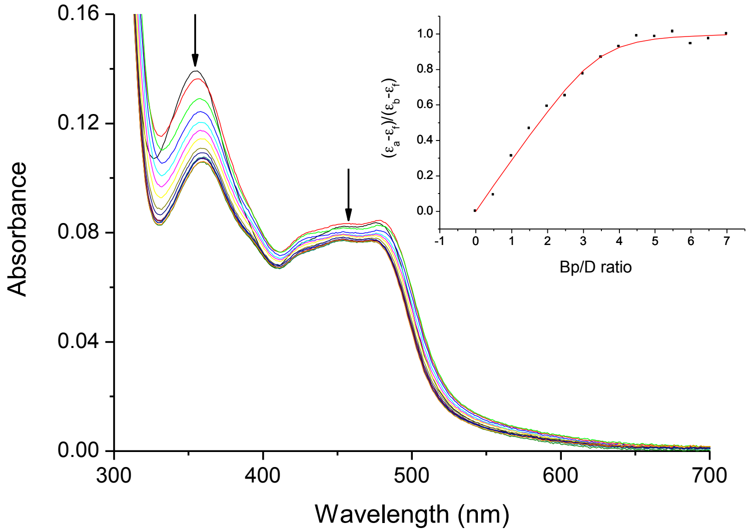

Figure S6. Changes in the UV/Visible spectrum of **Ru-C$_3$-2Nap-4NO$_2$** (6.5 μM) upon addition of st-DNA (0 – 45.5 μM base pairs) in 10 mM phosphate buffer, at pH 7. Inset: Plot of $(\varepsilon_a\text{-}\varepsilon_f)/(\varepsilon_b\text{-}\varepsilon_f)$ vs. [DNA] and the corresponding non-linear fit. (Bp/D = base pair to complex).

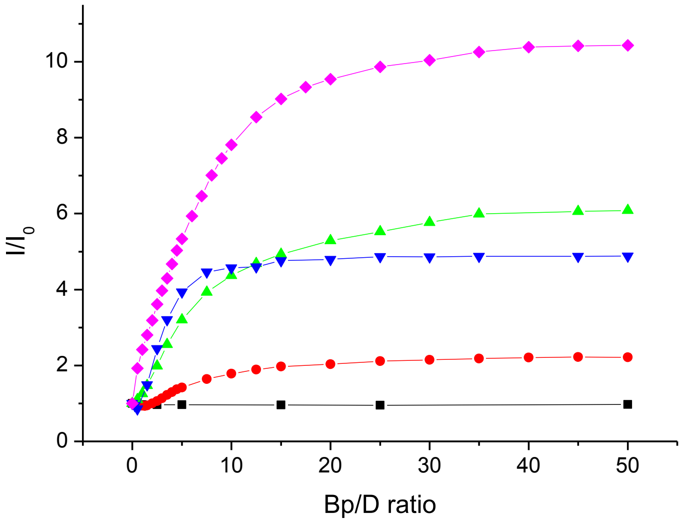

Figure S7 Relative change in the integrated emission intensity of **Ru-C$_3$-Nap-4NO$_2$** (●), **Ru-C$_5$-Nap-4NO$_2$** (▲), **Ru-C$_3$-2Nap-4NO$_2$** (▼), **Ru-C$_5$-2Nap-4NO$_2$** (◆) and **Ru-1** (■) upon addition of st-DNA in 10 mM phosphate buffer, at pH 7. (λ_{ex} 450 nm). (Bp/D = base pair to complex).

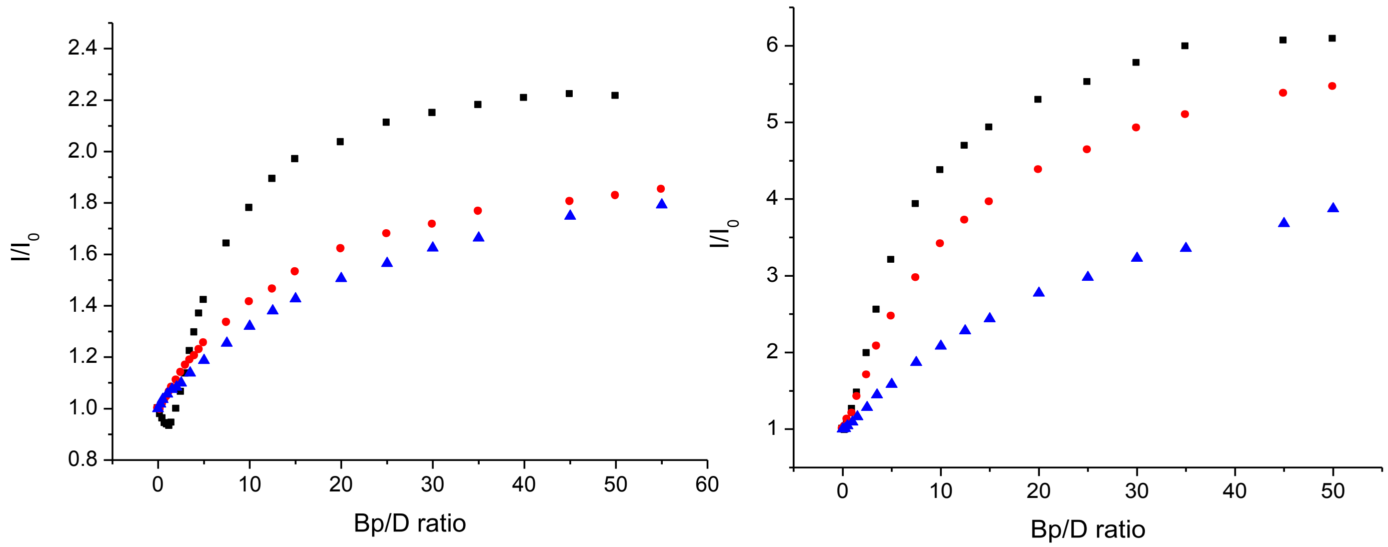

Figure S8 Relative changes in the emission of 6.5 μM (a) **Ru-C$_3$-Nap-4NO$_2$** and (b) **Ru-C$_5$-Nap-4NO$_2$** with increasing concentration of st-DNA (0 – 325 μM), and (b) in 10 mM phosphate buffer (■), 10 mM phosphate buffer + 50 mM NaCl (●) and 10 mM phosphate buffer + 100 mM NaCl (▲).

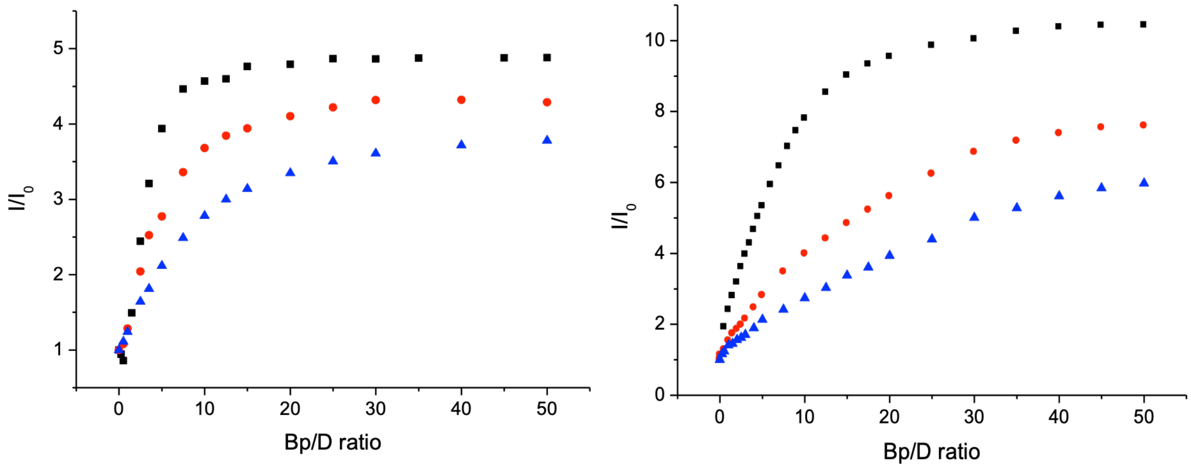

Figure S9. Relative changes in the integrated emission intensity of 6.5 μM (a) **Ru-C$_3$-2Nap-4NO$_2$** and (b) **Ru-C$_5$-2Nap-4NO$_2$** with increasing concentration of st-DNA (0 – 325 μM), in 10 mM phosphate buffer (■), 10 mM phosphate buffer + 50 mM NaCl (●) and 10 mM phosphate buffer + 100 mM NaCl (▲). (λ_{ex} 450 nm). (Bp/D = base pair to complex).

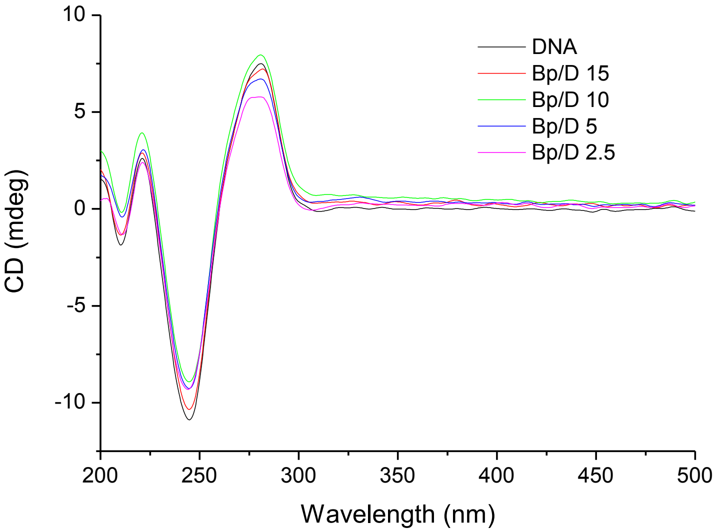

Figure S10 Circular dichroism spectra of ct-DNA (150 μM) in 10 mM phosphate buffer, at pH 7, in the absence and presence of **Ru-1** at varying ratios. (Bp/D = base pair to complex).

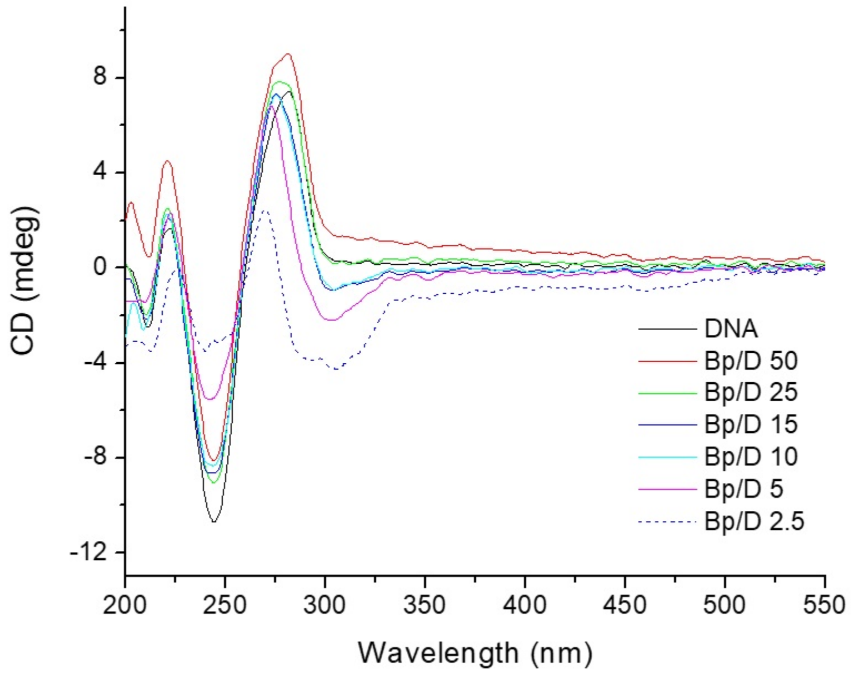

Figure S11. Circular dichroism spectra of st-DNA (150 μM) in 10 mM phosphate buffer, at pH 7, in the absence and presence of **Ru-C$_3$-2Nap-4NO$_2$** at varying ratios.

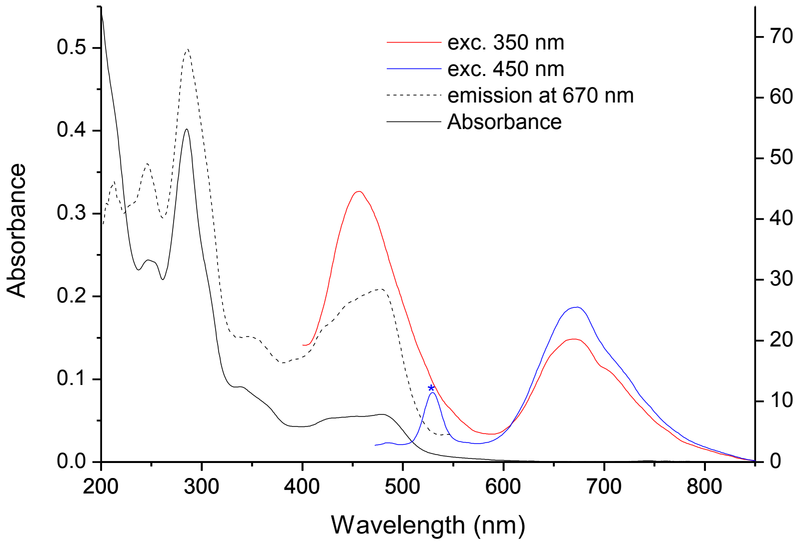

Figure S12 UV/Visible, excitation and emission spectra of 6.5 μM **Ru-C$_5$-2Nap-3NO$_2$** in 10 mM phosphate buffer, at pH 7. The water Raman band is denoted by *.

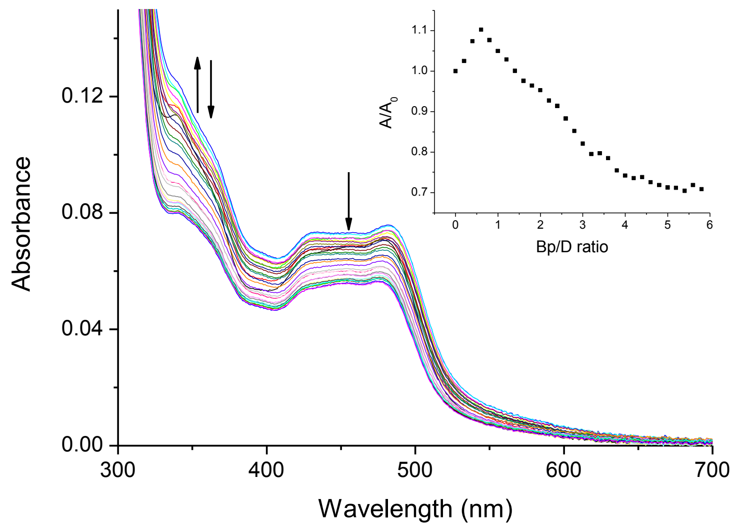

Figure S13 Changes in the UV/Visible spectrum of 6.5 μM **Ru-C$_5$-2Nap-3NO$_2$** in 10 mM phosphate buffer, at pH 7 upon addition of st-DNA (0 – 37.7 μM). (Bp/D = base pair to complex).

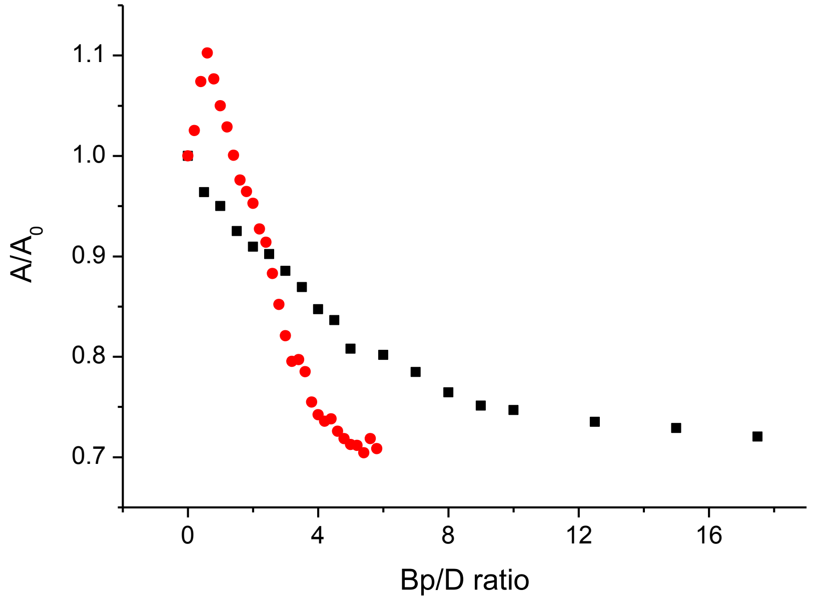

Figure S14 Changes in the 1,8-naphthalimide absorption of 6.5 μM **Ru-C$_5$-2Nap-4NO$_2$** (■) and **Ru-C$_5$-2Nap-3NO$_2$** (●) in 10 mM phosphate buffer,at pH 7 upon addition of st-DNA (Bp/D = base pair to complex).

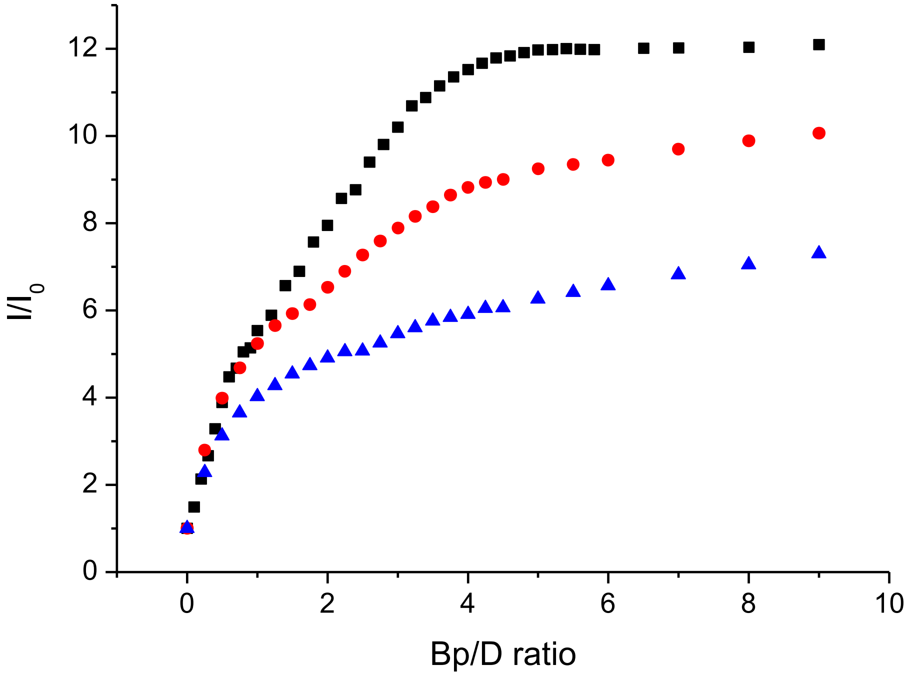

Figure S15 Relative changes in the emission of **Ru-C$_5$-2Nap-3NO$_2$** (6.5 μM) with increasing concentration of st-DNA (0 – 58.5 μM), in 10 mM phosphate buffer (■), 10 mM phosphate buffer + 50 mM NaCl (●) and 10 mM phosphate buffer + 100 mM NaCl (▲). Bp/D = base pair to complex).

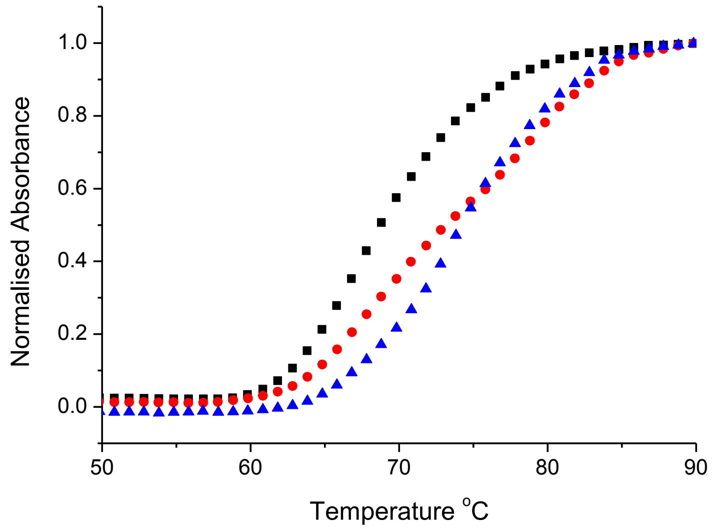

Figure S16 Thermal denaturation curves of st-DNA (150 μM) in 10 mM phosphate buffer, pH 7, in the absence (■) and presence of **Ru-C$_5$-2Nap-3NO$_2$** at P/D ratio of 10 (●) and 5 (▲).

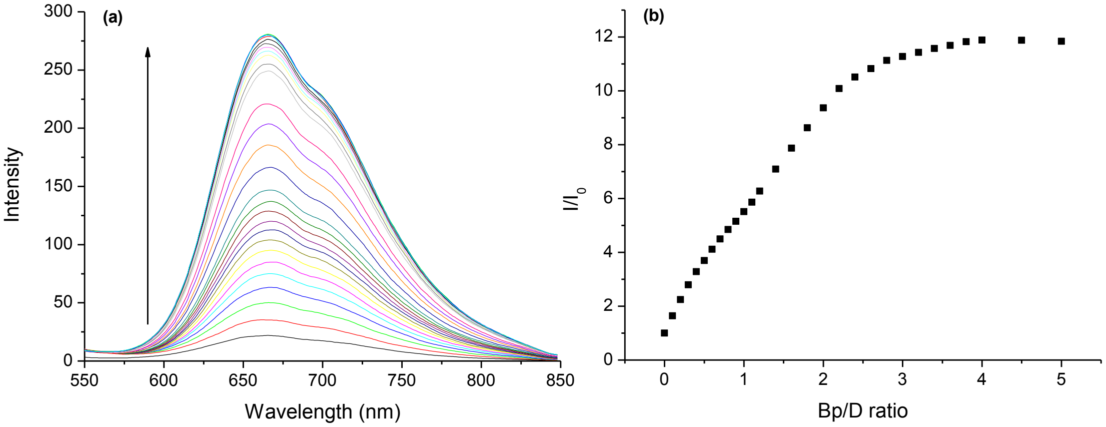

Figure S17 (a) Changes in the MLCT emission of **Ru-C$_5$-2Nap-3NO$_2$** (6.5 μM) (λ_{ex} 450 nm) upon addition of [poly(dG-dC)]$_2$ (0 – 32.5 μM base pairs) in 10 mM phosphate buffer, at pH 7. (b) The relative change in integrated emission intensity.

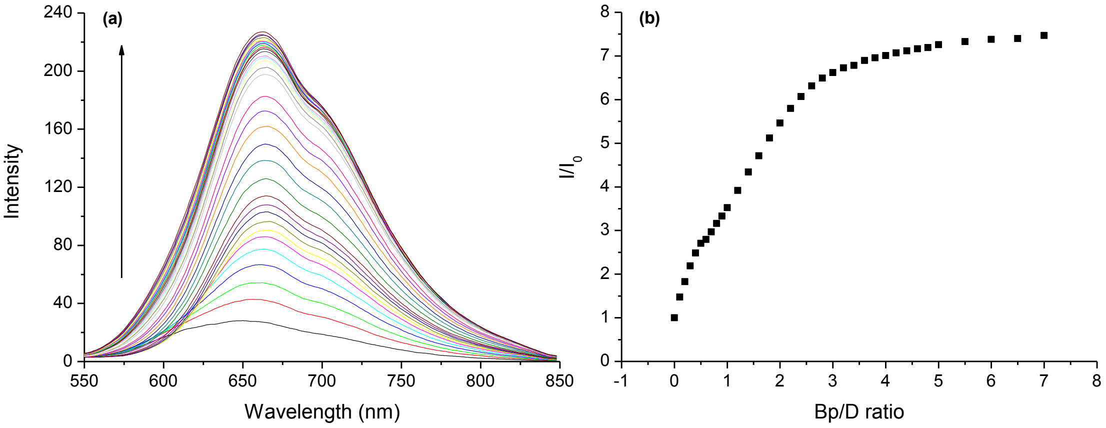

Figure S18 (a) Changes in the MLCT emission of **Ru-C$_5$-2Nap-3NO$_2$** (6.5 μM) (λ_{ex} 450 nm) upon addition of [poly(dA-dT)]$_2$ (0 – 45.5 μM base pairs) in 10 mM phosphate buffer, at pH 7. (b) The relative change in integrated emission intensity.
